# Spatial transcriptomics identifies distinct domains regulating yield-component traits of the wheat ear

**DOI:** 10.1126/sciadv.aed1407

**Published:** 2026-06-17

**Authors:** Yue Qu, Cong Tan, Liujing Yang, Marianna Pasquariello, Abdul Kader Alabdullah, Shiyu Sun, Munir Iqbal, John Salamon, Scott A. Boden

**Affiliations:** ^1^ School of Agriculture, Food and Wine, Waite Research Institute, Adelaide University, Glen Osmond, SA 5064, Australia.; ^2^ Key Laboratory of Genomics, Ministry of Agricultural, BGI Bioverse, BGI Research, Shenzhen 518083, China.; ^3^ College of Life Sciences, University of Chinese Academy of Sciences, Beijing 100049, China.; ^4^ Crop Genetics Department, John Innes Centre, Norwich Research Park, Norwich NR4 7UH, UK.; ^5^ Council for Agricultural Research and Economics, Research Centre for Genomics and Bioinformatics, Via San Protaso 69, Fiorenzuola d’ Arda (PC) 29017, Italy.; ^6^ South Australian Genomics Centre, SAHMRI, North Terrace, Adelaide, SA 5000, Australia.

## Abstract

Cereals form complex, highly ordered inflorescences composed of grain-producing florets that form within spikelets. Wheat spikelets are arranged in alternating rows along a central rachis, in a pattern determined during early development. While several genes controlling spikelet formation have been identified, it is still unclear how they interact to regulate inflorescence morphology and the development of supporting structures, including meristems and the rachis. Here, we used spatial transcriptomics to investigate the transcriptional landscape of a developing wheat inflorescence. We identified two spatially distinct regions that regulate spikelet architecture, including a primordium region characterized by *RAMOSA2* and a boundary region that expresses *ALOG1* and bract suppressors. Developmental assays indicate that spikelets differentiate from meristems and are accompanied by formation of the central vasculature that expresses spikelet number and fertility genes. The combined spatial transcriptome and genetic data reveal key regulators of spikelet development, including target genes for improving spikelet number and yield.

## Teaser

Mapping gene activity in the wheat ear reveals domains that regulate spikelet number and yield potential.

## INTRODUCTION

The inflorescence of bread wheat (*Triticum aestivum*), known as the spike, is a complex and highly ordered structure composed of spikelets, which form grain-bearing florets. As grain number is a key yield determinant, the number of spikelets and fertile florets that form on a spike is crucial for global food security, especially because wheat accounts for 20% of the calories and protein consumed worldwide (*1*). The early inflorescence stages of double ridge (DR) and lemma primordium (LP) mark a pivotal developmental phase that establishes the foundation for the final spike architecture and potential grain number (*2*, *3*). During the DR stage, the inflorescence meristem differentiates to generate distinct lateral meristems destined to become spikelets, marking the transition from vegetative to reproductive growth (*4*). At the LP stage, individual spikelets differentiate further to initiate lemma primordia and floret meristems, setting the groundwork for floret formation and grain production (*4*).

Given their importance for grain production, many studies have sought to identify genes that regulate the number, arrangement, and fertility of spikelets that form on the wheat spike. Genes including *Photoperiod-1* (*Ppd-1*), *FLOWERING LOCUS T2* (*FT2*), *PHOTOPERIOD-1–DEPENDENT bZIP TRANSCRIPTION FACTOR1* (*PDB1*), *CONSTANS-LIKE5* (*COL5*), *ZINC FINGER1* (*ZF1*), and *TERMINAL FLOWER1* (*TFL1*) influence spikelet number by modulating the strength of the flowering signal or the timing of spikelet termination, with many expressed during the DR or LP stage (*2*, *3*, *5*–*8*). Spikelet architecture genes—such as *ALOG1*, *DUO1*, *TEOSINTE BRANCHED1* (*TB1*), *HOMEOBOX DOMAIN-2* (*HB-2*), and *WHEAT FRIZZY PANICLE* (*WFZP*)—have been identified by analyzing genotypes that form supernumerary spikelets or branched spikes, and they are similarly expressed during these early developmental stages (*3*, *9*–*13*). Key regulators of spikelet fertility include MADS-box and homeodomain leucine zipper class I transcription factors, such as SHORT VEGETATIVE PHASE1/2 (SVP1/2) and GRAIN NUMBER INCREASE1 (GNI1), which influence either the fertility of basal spikelets or the survival of florets within each spikelet (*14*–*18*). While these studies highlight the emerging capabilities of wheat developmental genetics, it is sobering that each gene was identified individually through lengthy studies reliant on unique natural or induced variant alleles, which are often dominant because of the redundancy of wheat’s hexaploid genome. This shortcoming highlights the need for new approaches that enable broader and more rapid assessment of genes influencing wheat inflorescence development.

Spatial transcriptomics is an emerging and rapidly advancing technology that enables unprecedented spatial resolution of gene expression within intact tissue structures, overcoming limitations of conventional transcriptome approaches that analyze bulk tissue samples (*3*, *18*, *19*). Recent spatial transcriptomics studies have demonstrated the potential of this approach to enhance our understanding of cereal development; in maize, spatial transcriptomics identified distinct meristem subtypes during ear development and uncovered spatially specific expression of key regulatory genes (*20*). Similarly, hybridization- and transcriptome-based spatial platforms have been used to investigate transcriptional networks underlying wheat inflorescence development, which supported characterization of transcription factors (e.g., bZIP19, BRASSINAZOLE RESISTANT1 [BZR1], LEAFY [LFY], and SVP1/2) that regulate traits such as spike length, spikelet number, and fertility (*21*–*24*). These advances highlight spatial transcriptomics’ potential to elucidate previously inaccessible gene expression patterns that control early spike development, especially in wheat, where next-generation sequencing can resolve homeologous transcripts of the three genomes (A, B, and D). Clarifying these expression patterns could help bridge existing knowledge gaps about spikelet formation, ultimately informing targeted wheat breeding strategies designed to optimize inflorescence architecture and enhance grain yield potential.

In this study, we used Stereo-seq (spatial enhanced-resolution omics sequencing) to generate a spatially resolved, single-cell transcriptome atlas of the wheat inflorescence at the DR and LP developmental stages. These stages represent a critical developmental window when the spikelet number and architecture are set, making them ideally suited to capture regulatory programs that determine a key yield trait (*6*). This high-resolution approach revealed previously unidentified spatial patterns of gene expression associated with spikelet initiation and differentiation. Our analysis highlights *RAMOSA2* (*RA2*), a gene identified through forward genetics as a determinant of spikelet number and yield-related traits, thereby bridging spatial transcriptomics with targets from wheat improvement. By integrating spatial expression maps with functional genetic insights and identifying domains that control spikelet number and arrangement, our findings establish a framework to dissect the regulatory basis of spikelet development and provide a resource for targeted yield improvement in wheat.

## RESULTS

### High-resolution spatial transcriptome profiling of wheat inflorescences using Stereo-seq

**Fig. 1.**
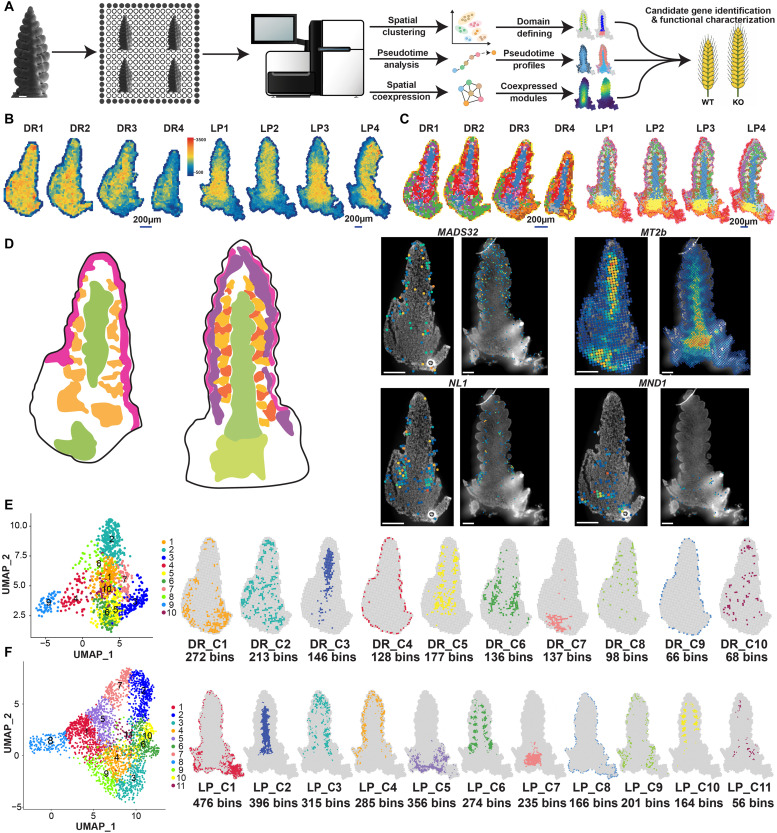
Spatial transcriptomics workflow and identification of transcriptional domains in early wheat inflorescence development. (**A**) Schematic overview of the spatial transcriptomics pipeline that informs candidate gene identification and functional validation. KO, knockout. Illustration adapted from genomesequencer-2 icon by DBCLS (https://togotv.dbcls.jp/en/pics.html); licensed under CC BY 4.0 Unported (https://creativecommons.org/licenses/by/4.0/). (**B**) Spatial transcript count maps and (**C**) spatial binning resolution optimization across four biological replicates of DR (DR1 to DR4) and LP (LP1 to LP4) stages. Heatmaps indicate UMI counts per bin, and representative spatial maps of DR (bin40) and LP (bin50) show selected bin sizes based on a balance between spatial resolution and transcript capture (see table S1). Images of DR (bin40) and LP (bin50) are also included in fig. S2 to provide a comparison to other bin sizes. Scale bars, 200 μm. (**D**) Schematic of spatial gene expression patterns that overlap with key inflorescence tissues: meristems (pink and purple), rachis and basal vasculature (dark and light green), and spikelets (light and dark orange). Gene expression patterns of *MADS32*, *MT2*, *NL1*, and *MND1* are localized to distinct regions of the inflorescence at the DR and LP stages, which overlap with previous MERFISH data (images for all four replicate sections shown in figs. S3, S5, S6, and S9 to S12). Scale bars, 200 μm. (**E** and **F**) Unsupervised clustering of DR (E) and LP (F) transcriptomes, with UMAP projection of spatial bins identifying 10 (DR_C1 to DR_C10) and 11 (LP_C1 to LP_C11) transcriptionally distinct clusters. The number of bins in each cluster is indicated [(E) and (F)].

To investigate cell differentiation and cell fate determination associated with wheat spikelet development, we performed spatial transcriptomic profiling for inflorescences at the DR and LP stages. Developing spikes from the elite cultivar Mace were flash-frozen in optimal cutting temperature resin, sectioned longitudinally at 12 μm, and mounted onto 1-cm by 1-cm STOMics Stereo-seq chips. These sections were subjected to a workflow that included nucleus and cell wall staining, analysis of tissue integrity, tissue permeabilization, cDNA synthesis, and library generation and sequencing, in preparation for the downstream analyses (Fig. 1A). We obtained high-quality spatial transcriptomes (1.76 billion high-confidence reads) for four sections of the DR and LP stages, with means of 48,178 and 53,373 genes detected across the four replicate sections for DR and LP, respectively, representing 89.6 and 96.3% of expressed transcripts detected using bulk RNA sequencing (RNA-seq) analysis of the same stages (*3*). The relative ranking of transcripts based on their abundance was also well conserved between the spatial transcriptome and bulk RNA-seq datasets for the DR (Spearman’s ρ = 0.67, *P* < 2.2 × 10^−16^) and LP (Spearman’s ρ = 0.64, *P* < 2.2 × 10^−16^) stages. The high-resolution stained images and sequencing data of the four sections for each stage were used to perform spatial clustering at different bin sizes (Fig. 1, B and C; table S1; and figs. S1 and S2). bin40 and bin50, which approximate the size of a single cell in the developing wheat inflorescence (*25*), defined regions corresponding to key structural features (e.g., spikelet primordia, rachis, inflorescence meristem, and lateral meristems) at the DR and LP stage, respectively. The average number of genes detected per pseudocell ranged from 590 to 1004 in DR samples and 768 to 857 in LP samples (table S1). As the clusters identified at these bin sizes were consistent with those detected using complementary approaches, these specifications were used for subsequent analyses (*21*–*23*).

We validated the spatial gene expression data by comparing our results to the localization of a selected and random set of genes obtained in other studies using independent assays. Homeologous transcripts for key marker genes including *NECK LEAF1* (*NL1*), *MADS32*, *METALLOTHIONEIN2* (*MT2*), *MANY NODED DWARF1* (*MND1*), *ALOG1*, *LAX PANICLE1* (*LAX1*), and *VEGETATIVE TO REPRODUCTIVE TRANSITION2* (*VRT2*) exhibited spatially distinct expression patterns consistent with known tissue-specific localization during inflorescence development (Fig. 1D and figs. S3 to S14) (*3*, *21*, *22*). For example, *NL1* and *ALOG1* transcripts localized to the lower region of the lateral meristem that subtends spikelet primordia, while *MADS32* and *LAX1* transcripts localized to the initiating spikelet primordia and the base of differentiated spikelets at the DR and LP stages, respectively. *MT2* transcripts localized to the central vasculature of the developing rachis at both stages, matching the phloem-specific expression of rice orthologs, and *MND1* transcripts were detected in vasculature at the inflorescence base, with a stronger signal detected at the DR stage than the LP stage. Our data, therefore, align closely with spatial patterns of gene expression detected using complementary approaches across diverse inflorescence tissues (*3*, *21*, *22*).

### Spatial clustering defines distinct transcriptional domains during early inflorescence development

To investigate transcriptional domains in the developing inflorescence, we performed unsupervised clustering analysis using all four sections for each of the DR and LP stages. This analysis detected 10 clusters at the DR stage and 11 at the LP stage, which resolved into spatially distinct regions on the basis of their uniform manifold approximation and projection (UMAP) (Fig. 1, E and F). Two DR clusters, designated the spikelet initiation (DR_C5) and leaf ridge (DR_C6) clusters, located to regions lateral to the main axis where spikelet and leaf primordium form, respectively. Similarly, two distinct LP clusters, termed spikelet primordia (LP_C10) and spikelet boundary (LP_C6), were arranged distichously along the central axis that aligned with developing spikelets. We also detected domains at both stages that were characteristic of the central rachis and basal vasculature of the inflorescence (DR_C3 and DR_C7 and LP_C2 and LP_C7, respectively), and others localized to the apical inflorescence and lateral meristem regions (DR_C1, DR_C2, DR_C8, and DR_C10 and LP_C3, LP_C9, LP_C4, and LP_C11, respectively). The clusters were characterized by marker genes that exhibited strong expression in the representative bins (Fig. 1G, table S2, and figs. S15 to S17). The spikelet initiation cluster at the DR (DR_C5) stage was represented by homeologous transcripts encoding NUCLEOLIN-like and AGONAUTE-like (AGO) proteins, large ribosomal subunits, and Heat Shock Protein 70 (HSP70) and HSP90; similar genes characterized the leaf ridge cluster (DR_C6), along with *ALOG-D1* that regulates spikelet architecture (*3*). At the LP, the spikelet primordium cluster (LP_C10) was represented by transcripts encoding an Aux/IAA protein (IAA13), SUCROSE SYNTHASE (SUS2), and a peptidyl prolyl cis-trans isomerase, while the spikelet boundary cluster (LP_C6) was characterized by genes encoding AGO, HSP70, HSP90, SQUAMOSA-promoter binding protein-like13 (SPL13), and a cytochrome P450 monooxygenase (CYP78-16) that influences organ size (*26*, *27*). The meristem regions were represented by transcripts encoding histones and ribosomal subunits, and these were maintained between DR and LP. The rachis and basal inflorescence regions of DR were characterized by transcripts encoding MT2, the aquaporin TIP1;1, a cytoplasmic glyceraldehyde-3-phosphate dehydrogenase (GAPDH) enzyme involved in glycolysis (GapC3), and homologs of the lectin protein EULS3 (*28*–*31*). Similar transcripts were detected in these regions at the LP, as well as transcripts encoding photosynthesis-related proteins (e.g., FERREDOXIN and CHLOROPHYLL A/B BINDING PROTEINS), which is consistent with the onset of rachis greening that occurs during this stage (*3*, *32*).

### Integration of spatial clusters and marker genes highlights domain conservation and differentiation between DR and LP

**Fig. 2.**
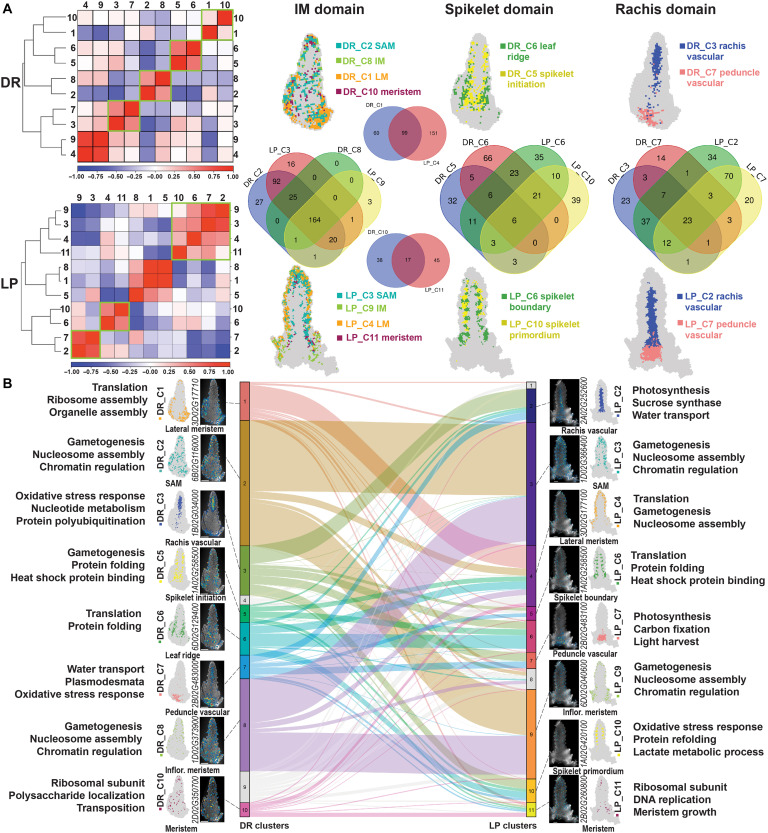
Identification of conserved and divergent spatial domains across inflorescence development. (**A**) Pairwise correlation heatmaps and hierarchical clustering define three major cluster bundles: inflorescence meristem (IM), spikelet, and rachis domains. Venn diagrams show the overlap of differentially expressed genes across stage-specific clusters within each domain. Cluster locations are highlighted in representative tissue sections. (**B**) Sankey plot illustrating the continuity and divergence of gene expression programs between clusters at the DR and LP stages. The prefix for the gene IDs is “*TraesCS*.” Selected marker genes and their enriched GO terms are shown alongside their spatial expression maps.

Next, we investigated the association between clusters within each stage, as well as the relationships between clusters of similar tissue types across stages (Fig. 2, A and B). Meristem domains (MDs) at the DR stage were composed of two pairs of correlated clusters, DR_C2 and DR_C8 and DR_C1 and DR_C10, which localized to apical and lateral regions of the developing inflorescence. Similarly, the well-correlated LP meristem clusters of LP_C3, LP_C4, LP_C9, and LP_C11 localized to the apex of both inflorescence and lateral meristems; overall, these data suggest that the position of meristems at apical and lateral regions of the developing inflorescence is conserved between DR and LP. The composition of marker genes and their ontologies within meristem clusters overlapped substantially across stages, indicating that their identity is well maintained between DR and LP, and meristems are sustained as cells differentiate into spikelet primordia. The meristem identity of these clusters is supported by their inclusion of genes known to control meristem size, with transcripts encoding FON2-LIKE CLE PROTEIN1/CLAVATA3 (FCP1/CLV3) and CLV1 localizing to inflorescence and lateral meristem cells at the DR and LP stages (tables S4 and S5 and fig. S18) (*33*–*37*). At the DR stage, *FCP1* and *CLV1* transcripts also localized to the leaf ridge cluster. The pair of clusters that defined the vasculature of the rachis and inflorescence base was strongly correlated at both the DR and LP stages, and a large proportion of transcripts that localized to the vascular regions at LP was detected in corresponding clusters at the DR stage (Fig. 2, A and B). Together, these results suggest that a key event of DR involves the establishment of central vasculature tissue that will eventually become the rachis and stem segments. The two spikelet-associated domains were represented by strongly correlated clusters of DR_C5 and DR_C6 at the DR stage and by LP_C6 and LP_C10 at the LP stage (Fig. 2, A and B). Relatively few genes were shared between these clusters within and across stages, indicating that at least two distinct groups of cells are required to coordinate spikelet formation, and the progression of spikelet development alters the transcriptional profile of these domains (Fig. 2, A and B, and table S3). Analysis of marker genes between DR and LP indicates that transcripts that localized to the spikelet primordia (LP_C10) were expressed in each of the spikelet- and vasculature-associated clusters at the DR stage, while those localizing to the spikelet boundary (LP_C6) associate mostly with the leaf ridge and rachis clusters of DR.

**Fig. 3.**
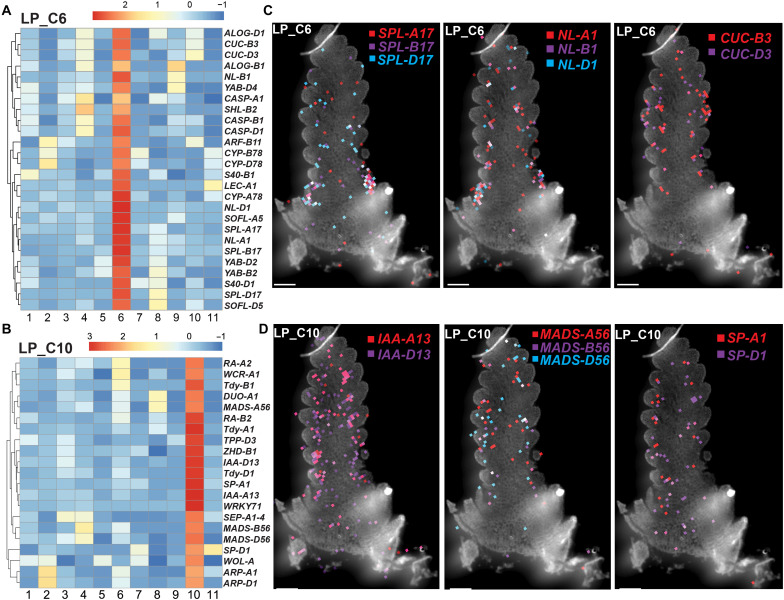
Examination of clusters localized to differentiated SDs. (**A** and **B**) Scaled expression (*z*-scores) of genes enriched in LP_C6 (A) and LP_C10 (B). LP_C6 represents a spikelet boundary cluster enriched for *SPL17*, *NL1*, and *CUC3*, while LP_C10 corresponds to spikelet primordium identity with enriched expression of *IAA13*, *GL10*, and *SP1*. (**C** and **D**) Spatial localization of representative genes from LP_C6 (C) and LP_C10 (D), showing distinct expression domains consistent with boundary and spikelet primordium regions, respectively. Image of *IAA13* also shown in fig. S18 along with the DR expression signal. Scale bars, 200 μm.

To further investigate the identity of the two spikelet-associated domains, we surveyed transcripts expressed in LP_C6 and LP_C10 that were represented by two or three homeologs (Fig. 3, A to D). All the genes enriched in LP_C6 and LP_C10 and other DR/LP clusters are shown in table S3. Support for the identity of LP_C6 as a spikelet boundary domain came from identification of genes that coordinate lateral organ boundary formation, such as *CUP-SHAPED COTYLEDON3* (*CUC3*) and *ALOG1* (*ALOG-B1* and *ALOG-D1*) (Fig. 3, A and C; table S3; and fig. S18) (*3*, *38*); ALOG1’s role in boundary formation helps lateral meristems form a single spikelet, rather than a spikelet pair (*3*). We also detected transcripts encoding NL1, SPL17, and S40, which suppress bract outgrowth or promote leaf senescence (*39*–*43*). Localization of these transcripts to the spikelet boundary is consistent with the suppression of leaf primordia that occurs on the abaxial side of developing spikelets and supports the model proposed in maize, whereby SPL17 (*Zm*TSH4) and NL1 (*Zm*TSH1) form a network to suppress bract growth and promote lateral meristem indeterminacy (*44*, *45*).

Transcripts encoding YABBY6 and the abaxial-associated transcription factor, KANADI, were enriched in the lower spikelet primordium cluster (LP_C6) (table S3 and fig. S18) (*11*, *46*–*47*). In contrast, transcripts of the class III homeodomain–leucine zipper gene *HB-2*, which promotes adaxial fate and paired spikelet formation when expressed at elevated levels, were enriched in the upper spikelet primordium cluster (LP_C10) (table S5 and fig. S18) (*11*). Together, these data indicate spatial separation of abaxial- and adaxial-promoting gene expression during early spikelet development. The LP_C6 cluster contained transcripts for all three homeologs of genes encoding a CASPARIAN STRIP MEMBRANE DOMAIN PROTEIN (CASP), which recruits lignin polymerization machinery for Casparian strip deposition in the endodermis; a SOB FIVE-LIKE (SOFL) protein involved in cytokinin-mediated development; and CYP78-16 (Fig. 3A, table S3, and fig. S18) (*27*, *48*). This cluster also contained transcripts for three homeologs of *TB1*, which regulates spikelet architecture in a dosage-dependent manner (table S5 and fig. S11) (*10*). The identity of LP_C10 as a spikelet primordium domain is supported by detection of *SEPELLATA1-4* (*SEP1-4*) transcripts, which multiplexed error robust fluorescence in situ hybridization (MERFISH) analysis identified as a glume marker (Fig. 3B and table S3) (*21*). Similarly, this cluster was enriched for genes that regulate spikelet and branch architecture in rice, barley, and wheat, such as *SHORT PANICLE1* (*SP1*), *MADS56*, and *DUO1* (Fig. 3, B and D, and table S3) (*9*, *49*, *50*). Three homeologous transcripts were identified for *IAA13*, which controls lateral organ boundary formation in an auxin-dependent manner, and an ACC oxidase (ACO2) that catalyzes the final step of ethylene biosynthesis (Fig. 3, B and D, and fig. S12) (*51*, *52*). The cluster also contained transcripts encoding proteins involved in sugar metabolism and transport, including trehalose-6-phosphate phosphatases (TPPs) that localize to comparable regions in maize, and homeologs of *TIE-DYED1* (*Tdy1*), which encodes a transmembrane protein that loads sugar into the phloem (Fig. 3B, table S3, and fig. S18) (*53*–*55*). Together with existing functional evidence, our data suggest that the alternating, distichous arrangement of spikelets along a wheat inflorescence depends on a reticulated lattice-like expression of at least two gene groups, which coordinate either spikelet development or boundary formation.

### Pseudotime analysis reveals developmental trajectories and tissue differentiation within the forming wheat inflorescence

**Fig. 4.**
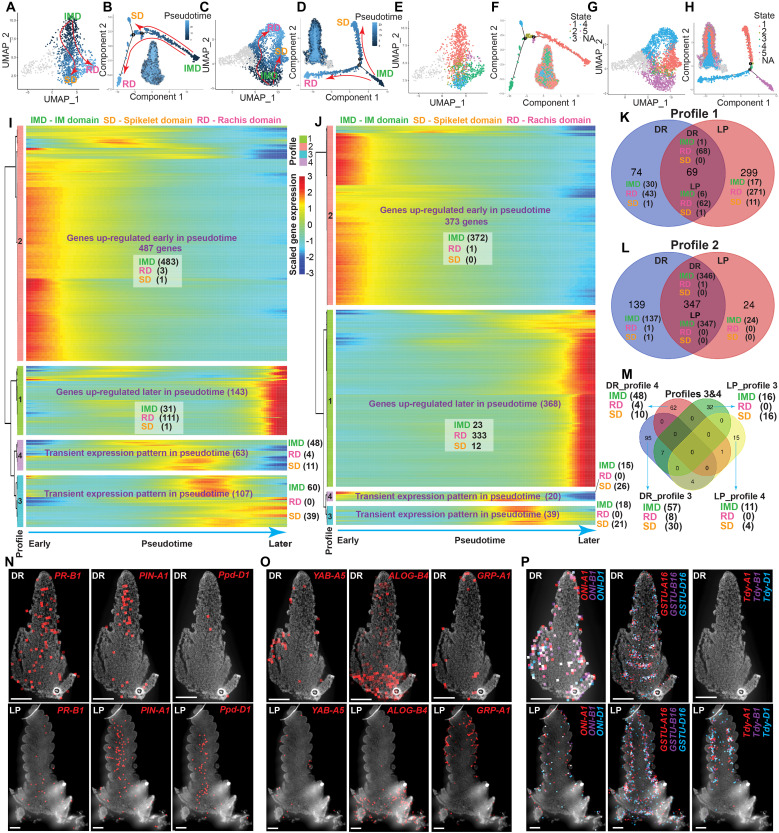
Pseudotime trajectory analysis reveals progressive differentiation of spatial domains in the wheat inflorescence. (**A** to **D**) Pseudotime inference using Monocle2 identifies two primary developmental trajectories in the DR [(A) and (B)] and LP [(C) and (D)] stages, originating from the inflorescence MD (IMD) and diverging toward the SD and RD. UMAPs are overlaid with pseudotime values [(B) and (D)] and domain annotations [(A) and (C)], revealing a gradual transition from undifferentiated to differentiated cell populations. (**E** to **H**) Trajectory-based clustering defines five pseudotime-associated cell states in DR (E) and LP (G). Tissue mapping of these states [(F) to (H)] demonstrates spatial coherence of developmental progression. (**I** and **J**) Heatmaps of pseudotime-dependent gene expression profiles in DR (I) and LP (J). Genes were grouped into four profiles on the basis of expression dynamics: profile 1, late up-regulated; profile 2, early up-regulated; profiles 3 and 4, transiently expressed at intermediate pseudotime. The number of genes assigned to each profile is indicated in brackets. (**K** to **M**) Venn diagrams comparing DR and LP gene sets for each pseudotime profile: late up-regulated (K), early up-regulated (I), and transiently expressed (M). Numbers of genes assigned to each profile that are expressed in inflorescence MD, RD, and SD are indicated. (**N** to **P**) Examples of shared and stage-specific genes for profile 1 (N), profile 2 (O), and profiles 3 and 4 (P) are indicated at the DR and LP stages. Images of *ALOG4* and *Ppd-1* at one stage are also shown in figs. S5 and S8, respectively, and non–genome-specific signals for *ONI-1* are shown in figs. S6 and S11. Scale bars, 200 μm.

To investigate tissue differentiation during early inflorescence development, we used Monocle2 to perform pseudotime analysis of cells at the DR and LP stages (*56*). Two major developmental trajectories were identified at each stage, with the LP displaying a clearer divergence in trajectory topology, consistent with tissue being more differentiated at the later stage (Fig. 4). The pseudotime trajectories originated from regions that overlap with meristem clusters and progressed toward those that associate with either spikelets or the rachis (Fig. 4, A and C). Trajectory-based clustering of pseudotime-associated cell states further supports the direction of these developmental trajectories (Fig. 4, B and D). DR and LP were each defined by five cell states, with three major states aligning with meristem, spikelet, or rachis regions (Fig. 4, E to H). Cells from earlier in the trajectory mapped to MDs, while more developmentally advanced cells localized to the rachis domain (RD) and spikelet domain (SD). Cells associated with intermediate segments of the trajectories localized to lateral meristem regions, suggesting that they contribute to spikelet formation. Next, we performed pseudotime-dependent expression analysis to identify genes that associate with early or late phases of the developmental trajectory. These genes were grouped into four expression profiles, depending on them being active during early (profile 2) or late phase (profile 1) or at intermediate phases (profiles 3 and 4) of the pseudotime continuum (Fig. 4, I and J, and table S4).

Genes encoding histone and ribosomal proteins are up-regulated early in the pseudotime at the DR and LP stages, consistent with meristems featuring early in the developmental trajectory (profile 2). Many of these genes shared the same profile at the DR and LP stages, supporting the view that meristems are sustained during early inflorescence development (Fig. 4, I to L, N, and O). Genes encoding MT2b, TIP1;1, GH3-4, PIN1, and the aquaporin PIP1;3, which localize to the rachis, were up-regulated late in the DR trajectory (profile 1) (Fig. 4, I, K, and N; table S6; and figs. S11 and S18). A similar gene set is expressed late in the LP trajectory, along with 299 unique transcripts that are enriched in the rachis, indicating more advanced differentiation of vascular tissue at the later stage (Fig. 4, J and K). The additional genes include *Ppd-D1* and *PDB1* that control flowering time and spikelet number and those encoding aquaporins (e.g., TIP2;3) and photosynthesis-related proteins (e.g., CAB1/2/3 and Psa and Psb proteins) (Fig. 4, K and N; table S6; and fig. S18) (*3*, *5*). Genes that regulate spikelet development (e.g., *ALOG1*, *CYP78*, *IAA13*, *SPL17*, *CUC3*, and *MADS32*) were expressed during transient phases of the trajectory (profiles 3 and 4) at both the DR and LP stages, with many showing stage- and profile-specific expression, suggesting that they perform temporally specific roles during spikelet formation (Fig. 4, M and P; table S6; and fig. S18). Together, these data indicate that spikelet development involves dynamic transcriptional changes in cells that differentiate from MDs during stages accompanied by rapid differentiation of central vascular tissue.

### Spatial coexpression network analysis identifies region- and stage-specific gene modules

**Fig. 5.**
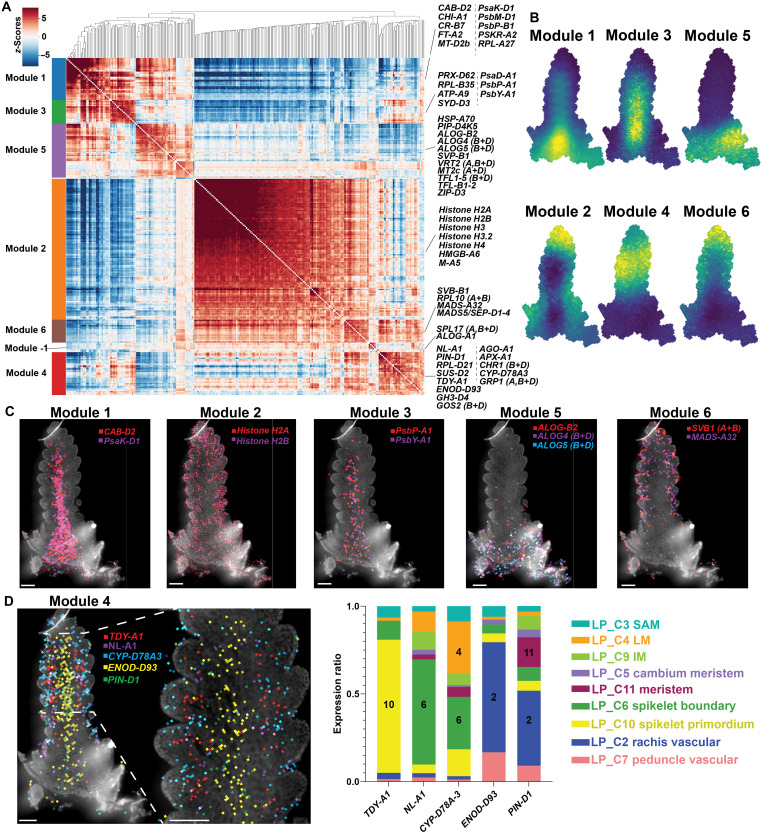
Spatial coexpression modules reveal transcriptional programs that distinguish developmentally diverse regions of the inflorescence. (**A**) The heatmap showing pairwise gene-gene correlations across spatial bins at the LP stage identifies six coexpression modules with distinct expression patterns. Modules enriched for known genes are annotated. (**B**) Module expression scores are mapped onto LP sections, revealing spatially distinct coexpression modules. (**C**) Expression of selected module-specific genes mapped to LP tissue images. Single gene images of *ALOG5*, *CAB-2*, and *MADS-32* are also shown in figs. S11 and S18 and Fig. 1D, respectively. (**D**) Spatial localization and cluster-specific expression of Module 4 genes. Left: Spatial expression of five representative Module 4 genes (*TDY-A1*, *NL-A1*, *CYP-D78A3*, *ENOD-D93*, and *PIN-D1*) mapped onto LP sections. These genes are enriched in the central region of the spike, corresponding to the most developmentally advanced spikelet primordia. Right: stacked bar plots show the proportional expression of each gene across LP spatial clusters, with numeric labels indicating the dominant contributing cluster. Module 4 genes are prominently expressed in LP_C10 (spikelet primordium), LP_C6 (spikelet boundary), and LP_C2 (rachis vascular). Scale bars, 200 μm.

Next, we performed coexpression network analyses to investigate spatially distinct gene regulatory modules within the inflorescence. Hierarchical clustering of gene expression correlations resolved the LP data into six discrete modules (Fig. 5A and table S7). Approximately 50 modules were identified among DR-expressed genes, many of which showed substantial overlap across different regions of the developing inflorescence (fig. S19). This pattern is consistent with a more dynamic transcriptional landscape at the DR stage compared to the LP stage, where tissue differentiation is more advanced. The six LP modules localized to regions aligned to either central and basal vascular regions [module 1 (M1), M3, and M5], meristems (M2), or spikelets (M4 and M6) (Fig. 5, B and C). The spikelet-associated modules localized to either the central region of the inflorescence, where spikelet formation is most advanced (M4), or the inflorescence apex where spikelets are less differentiated (M6). Module 4 comprised genes expressed in spikelet primordia and boundary regions, including *TDY1*, *NL1*, and *CYP-D78*, as well as vascular-associated genes such as *PIN1* and *ENOD93* (Fig. 5D and table S7) (*57*, *58*). This suggests that advanced spikelet development in the center of the spike involves coordinated regulation of genes across spikelet and vascular tissues. We also detected *NL-A1* and *CYP-D78A3* transcripts at the spikelet boundary during the DR stage and *ENOD-D93* and *PIN-D1* in vascular tissue at the DR stage, indicating that this gene network is involved in early phases of spikelet development (Fig. 1D, table S3, and fig. S5). Module 6 included spikelet primordium genes such as *MADS32* and the glume marker, *SEP1-4*, as well as genes from meristem clusters (*SVB-1* and *RPL10*), which reflects the ongoing differentiation of spikelets from meristematic tissue in this region (Fig. 5C and table S7).

The two spikelet modules correlated with a set of spikelet boundary genes (Module -1), including *ALOG1* and *SPL17* (Fig. 5A and table S7), which supports the identification of two discrete regions that contribute spikelet formation along the wheat inflorescence. Module 2 is composed of histone and ribosomal proteins representative of meristem regions, and it localized to apical and lateral regions of the inflorescence (Fig. 5, A and C, and table S7). M1 and M3 localized to vascular regions in the inflorescence that each contain transcripts encoding photosynthesis-related proteins; the separate networks detected for the center and base of the inflorescence indicate distinct identities for these vascular regions, potentially because the basal region will proceed to form stem internodes and the peduncle (Fig. 5, B and C). Genes unique to M1 included *FT2* (Fig. 5A, table S7, and fig. S18), which contributes to flowering time and termination of spikelet development (*6*, *59*). The inflorescence base was also represented by M5, which was enriched for transcripts encoding SVP1, VRT2, TFL1, and ALOG (ALOG2, ALOG4, and ALOG5) transcription factors (Fig. 5, B and C, and figs. S9, S11, and S18). This module aligns with regions of the inflorescence where spikelet development is often delayed, relative to the center, resulting in infertile spikelets; together with known roles for SVP1/2 and TFL in repressing early floral transition events, this region may help form a boundary between vegetative and reproductive zones of the developing inflorescence (*2*, *14*–*16*, *18*, *21*, *23*). *ALOG2/4/5* transcripts were also detected in rachis and spikelet regions at the DR stage, suggesting that they contribute to boundary formation within the inflorescence during early development (table S4 and fig. S5) (*3*, *60*). Overall, these modules provide insights into the progressive phases of spikelet development along the inflorescence, which may explain why spikelet fertility and branching phenotypes are more pronounced in the central region.

### *RA2* expression marks an SD that controls spikelet formation

**Fig. 6.**
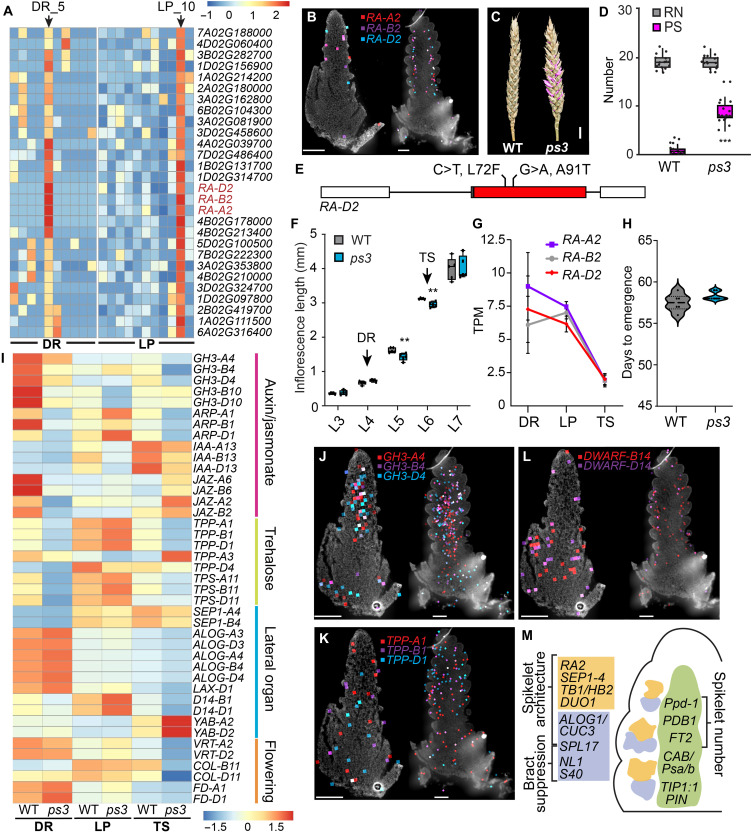
Analysis of developmentally conserved spikelet primordium genes identifies *RAMOSA2* (*RA2*) as a key regulator. (**A**) Heatmap showing genes coenriched in DR_C5 and LP_C10 clusters that define spikelet primordium domains at the DR and LP stages, including three homeologs of *RA2* (*RA-A2*, *RA-B2*, and *RA-D2*). Prefix to each gene ID is “*TraesCS*.” (**B**) Spatial expression profiles of *RA2* homeologs at the DR and LP stages. A single gene image comparing expression in each section to MERFISH (*21*) is shown in fig. S12. Scale bar, 200 μm. (**C** and **D**) *ps3* mutant spikes form secondary spikelets (pink) without altering rachis nodes (gray), relative to WT siblings. In (D), *n* = 16. Scale bar, 1 cm. (**E**) Schematic of *RA-D2* showing two missense mutations that promote paired spikelet development [white, UTR (untranslated region); red, exon 1]. (**F**) Analysis of inflorescence growth rate and developmental progression in WT (gray) and *ps3* (blue). In the box plot, each box is bound by the lower and upper quartiles, the central bar represents the median, and whiskers indicate the minimum and maximum values of five biological replicates. (**G**) Expression of *RA2* homeologs during early inflorescence development (data are the average ± SEM, three biological replicates). (**H**) Flowering time analysis of *ps3* (blue), relative to WT (gray), shown as a violin plot with individual data points (*n =* 6). (**I**) Heatmap displaying DETs in *ps3* inflorescences, relative to WT. (**J** to **L**) Localization of selected DETs at the DR and LP stages. An image of *GH3-A4* is shown in fig. S18. (**M**) Model illustrating how spatially distinct domains regulate spikelet architecture, bract suppression, and spikelet number in bread wheat. In (A) and (I), normalized expression values are scaled by row (*z*-score). Asterisks indicate statistical significance (**P < 0.01 and ***P < 0.001, Student’s *t* test).

The identification of two distinct clusters that align with spikelet initiation and development at the DR and LP stages indicated that at least two groups of cells, with different gene expression profiles, are required to direct the alternating, distichous arrangement of single spikelets along the wheat inflorescence. This model is supported by the role of *ALOG1*, which marks the spikelet boundary cluster at both stages and is required to restrict lateral meristem differentiation to a single spikelet (*3*, *60*). To further explore this model and the role of the spikelet initiation and spikelet primordium clusters in regulating spikelet architecture, we surveyed genes enriched in the spikelet initiation and spikelet primordium clusters of DR and LP, respectively, with a focus on homeologous triads that are expressed strongly in these domains. One triad includes homeologs of a gene encoding a lateral organ boundary domain containing a transcription factor that is homologous to RAMOSA2 from maize and Vrs4 from barley (*61*, *62*); we named the gene *RAMOSA2* (*RA2*). *RA2* is expressed exclusively in the spikelet initiation (C5) cluster of DR and predominantly in the spikelet primordium (C10) domain of LP (Fig. 6, A and B). We then asked whether any of the 256 paired spikelet–producing mutants identified from the Cadenza TILLING population contained mutations in a copy of *RA2* (*11*). Paired spikelets are a supernumerary spikelet structure characterized by the formation of a secondary spikelet immediately adjacent to and below the typical primary spikelet, and the absence of ALOG1 expression facilitates their development (*3*, *5*). Among class II paired spikelet–producing mutants (*11*) that form multiple secondary spikelets was *paired spikelet 3* (*ps3*; *CAD1591*) (Fig. 6C), which formed secondary spikelets at 45.9 ± 3.2% of rachis nodes (Fig. 6D). The paired spikelets formed predominantly in the center of the inflorescence, and scanning electron microscopy showed that the secondary spikelets emerged at the floret primordium and terminal spikelet (TS) stages, consistent with our previous analyses (fig. S20) (*5*, *10*, *11*). The *ps3* mutant contained a missense mutation (A91T) in the D genome copy of *RA2*, named *RA-D2* (*TraesCS3D02G093500*) (Fig. 6E); A91 residue is conserved among lateral organ boundary domain transcription factors and localizes to an α helix (α4) that stabilizes the homodimerization of RA2 required for DNA binding (*63*). The *ps3* line was crossed to *cv.* Cadenza four times to filter background mutations, and segregating BC_3_F_2_ populations were analyzed using exome capture sequencing and marker assays to show that the *ra-D2* missense allele associated significantly with paired spikelet production (fig. S21). This association was further verified using recombinant inbred lines, which excluded mutations linked to *ra-D2* (A91T) in exome capture sequence data of independent segregating BC_2_F_2_ families (fig. S21)*.* To support this role for *RA-D2* during spikelet development, we investigated an independent mutant line (*CAD0289*) that contained a missense mutation (L72F) that localizes to a neighboring α helix of a GAS (Gly-Ala-Ser) motif that is also required for homodimerization and stability of protein-DNA interactions (Fig. 6E) (*63*)*.* Like *ps3*, this *ra-*D2 missense (L72F) allele was only detected in paired spikelet–producing plants of segregating BC_2_F_2_ families and not in wild-type (WT) plants that expressed the reference allele (fig. S21).

To investigate the effect of these *ra-D2* missense alleles on inflorescence development, we analyzed inflorescence growth during early stages when spikelets and florets form. Inflorescence growth was delayed between DR and LP in plants of both mutant lines, relative to WT siblings (Fig. 6F and fig. S22). The delay between these stages is consistent with expression of *RA-D2* and its homeologs, *RA-A2* and *RA-B2* (*TraesCS3A02G093200* and *TraesCS3B02G108500*), peaking during the DR and LP stages before declining at the TS stage, with no transcripts detected at later stages (Fig. 6G). The delay was specific to these stages, as mutant plants reached TS and flowered at the same time as WT, consistent with there being no difference in rachis node number between WT and mutant genotypes (Fig. 6, F and H, and fig. S22). To assess whether the developmental delay observed in *ra-D2* mutants influenced yield-related traits, we introgressed the *ps3* allele into elite cultivars and evaluated grain production under glasshouse and field conditions. In the glasshouse, *ps3* plants of both Mace and Rockstar elite backgrounds produced significantly more grains per plant and greater total grain weight than WT siblings, which was associated with an increased frequency of paired spikelets (fig. S23, A to C). Similar effects were also observed in field-grown plants (fig. S23, D to F).

Next, we performed RNA-seq transcriptome analysis during early developmental stages to investigate molecular processes underlying paired spikelet development in *ps3*. We detected 763 differentially expressed transcripts (DETs) at the DR stage, of which 84.5% were down-regulated in *ps3*, relative to WT, while 368 and 792 DETs were detected at the LP and TS stages, respectively, with similar proportions of up- and down-regulated genes (table S8). At the DR and TS stages, down-regulated transcripts were enriched for processes related to trehalose metabolism, consistent with transcriptome changes detected in the barley *vrs4* mutant (Fig. 6I and fig. S24) (*62*). Down-regulated transcripts included homeologs for three genes encoding TPPs (TPP1, TPP3, and TPP4), and an α-trehalose-phosphate synthase (TPS11). Other down-regulated transcripts in *ps3* at the DR stage include those encoding a SEPALLATA-like MADS box transcription factor (SEP1-B4) and proteins involved in auxin and jasmonate signaling processes, such as Gretchen Hagen3 (GH3) acyl acid amido synthetases (e.g., GH3-8) and jasmonate ZIM-domain proteins (e.g., JAZ2 and JAZ6). Enrichment of genes involved in auxin signaling pathways was also detected among down-regulated transcripts at the LP and TS stages, including those encoding auxin/indole-3-acetic acid proteins (AUX/IAA; IAA13 and IAA26), GH3-1, GH3-4, an auxin repressed protein (e.g., ARP1), and a small auxin up-regulated RNA (SAUR). Transcripts down-regulated in *ps3* at the TS stage were also enriched for processes related to lateral organ formation and strigolactone signaling, such as *ALOG3*, *ALOG4*, *LAX1*, *WHEAT ORTHOLOGUE OF APO1* (*WAPO1*), and *DWARF14* (*D14*) (*3*, *64*, *65*). Similarly, genes involved in flowering and spikelet development [e.g., *VRT2* (*14*–*16*, *18*), *FLOWERING LOCUS D1* (*FD1*) (*66*), and *CONSTANS-like 10* (*COL10*) (*67*)] were significantly down-regulated in *ps3* at the TS stage, relative to WT, while YABBY2 transcripts were significantly higher in *ps3*. Curiously, no transcripts that are expressed in spikelet boundaries and proposed to act antagonistically to *RA2* were significantly misregulated in *ps3*, relative to WT; nonetheless, all homeologs for *SPL17*, *NL1*, *LG2*, *CUC3*, and *ALOG1* were moderately higher in *ps3* at the DR stage, relative to WT, and lower at the TS stage, indicating that delayed differentiation of the spikelet meristem did influence genes expressed in the neighboring boundary region (Fig. 6I and table S9). Together, these results indicate that secondary spikelet formation in *ps3* involves misregulation of processes involved in trehalose and hormone metabolism and signaling, as well as flowering and lateral organ formation.

To further investigate the DETs, we used the spatial transcriptome data to localize their expression within developing inflorescences at the DR and LP stages (Fig. 6, J and K, and tables S3 to S5). Among DETs detected in *ps3* at the DR stage, 24.7% (107 of 434) of genes localized to the spikelet-associated clusters, while 26.7% (116 of 434) and 25.1% (109 of 434) were from the meristem and rachis clusters, respectively. At the LP stage, a similar proportion of DETs localized to the spikelet-associated (21.7%; 54 of 249) and rachis-associated (26.1%; 65 of 249) clusters, while fewer were detected in meristem regions (14.1%; 35 of 249). Most of the differentially expressed *TPPs* (six of seven genes) and *TPS-B11* localized to the spikelet primordium cluster at the LP stage, while there was no shared location for these transcripts at the DR stage (Fig. 6K and tables S4 and S8). DETs encoding proteins involved in auxin signaling and metabolism, such as GH3-4, ARP1, and IAA13, located to spikelet primordium and meristem clusters, while *LAX1* and *SEP1-4* transcripts were enriched in the spikelet primordium (Figs. 2F and 5J and tables S3 to S5 and S8). Transcripts encoding YABBY transcriptions factors (e.g., YAB2), as well as SPL17 and D14, were divided between the spikelet primordium and boundary cells (Figs. 2D and 5L, table S8, and fig. S14). Transcripts encoding proteins involved in flowering (COL10) and auxin transport (e.g., PIN10) were present in rachis and peduncle tissue, while *VRT2*, *ALOG3*, and *ALOG4* transcripts localized to the inflorescence base (Fig. 4I, figs. S5 and S9, and table S8). Together with the bulk tissue RNA-seq analysis, the spatial transcriptome reveals that many transcripts differentially expressed in *ps3* localize to spikelet-associated clusters.

## DISCUSSION

Our Stereo-seq transcriptome analysis provides a high-resolution, state-of-the-art spatiotemporal map of gene expression during spikelet initiation and differentiation in the wheat inflorescence. By localizing transcripts within intact inflorescence sections at the DR and LP stages, we identified distinct gene expression domains that correspond to developing spikelets, apical and lateral meristems, and the central vascular regions of the rachis and inflorescence base. Together with functional knowledge of genes that localize to these domains (*3*, *5*, *9*–*11*, *14*–*16*, *18*, *40*), the spatial transcriptome map provides insights into how gene expression is coordinated across different cell types to determine the architecture, number, and fertility of spikelets that form along the inflorescence (Fig. 6M).

In regions that aligned with developing spikelets, we identified two distinct clusters at the DR and LP stages that were arranged in an alternating distichous pattern flanking the central rachis. One of these clusters specifies an upper group of cells that form spikelet primordia, characterized by expression of *RA2*, *SEP1-B4*, *IAA13*, and *DUO1*, while the lower cluster marks a boundary region, enriched for transcripts of *SPL14/17*, *NL1*, *S40*, and *ALOG1* that perform roles in bract suppression and spikelet development (*3*, *39*–*45*). We propose that these two groups of cells function synergistically to coordinate the formation of short lateral branches, each composed of a single spikelet that lacks a subtending bract, and their reiterative expression pattern along the inflorescence facilitates the sequential formation of multiple rachis nodes (Fig. 6M). In this model, spikelet boundary cells perform two distinct roles in wheat. First, they are likely to suppress bract outgrowth through the activity of *NL1*, consistent with findings from corresponding mutants in barley, rice, and maize (*41*, *44*, *68*, *69*). Second, they promote formation of a single spikelet—rather than a spikelet pair—in the adjacent primordium, supported by evidence that *ALOG1* functions non–cell-autonomously to suppress paired spikelet development in wheat and barley (*3*, *60*). The proposed role that boundary cells perform in generating multiple rachis nodes through their reiterative expression pattern along the inflorescence is supported by *spl14* and *spl17* wheat mutants forming substantially fewer spikelets than WT siblings, with many basal spikelets aborting their development (*40*). The localization of three *RA2* homeologs to the spikelet primordium cluster at the DR and LP stages indicates that it is a key gene driving spikelet initiation and development. This role is supported by *ra-D2* mutants forming paired spikelets, which form when differentiation of a lateral meristem into a spikelet is delayed (*5*, *10*)—the role of RA2 in spikelet differentiation is also supported by studies in maize and barley (*61*, *62*). Further spatial transcriptomic analyses of paired spikelet–producing mutants, including *ps3*, could be undertaken in future studies to determine how mutations in key spike architecture genes alter the spatial expression of genes that promote spikelet development. Together with the functional characterization of other paired spikelet mutants, we propose that genes expressed in the upper spikelet primordium and lower boundary regions act cooperatively to determine spikelet architecture, facilitating the formation of single spikelets on opposite sides of the central rachis in an alternating phyllotaxy (Fig. 6M) (*3*, *5*, *9*–*11*). A defining feature of the wheat inflorescence is that central spikelets produce more fertile florets than those at the apex or base, which is associated with spikelets developing earlier in the center than the top or bottom (*17*, *70*, *71*). The spatial transcriptome data and gene coexpression network analysis provided valuable insights into the molecular basis of asynchronous spikelet development, with separate modules identified for the center, apex, and base. The two modules representing the center and apex included genes from spikelet and rachis tissues; however, the apical module was enriched for transcripts corresponding to an earlier phase of the pseudotime trajectory compared to those observed in the central module. These results suggest that apical spikelets share a similar transcriptome profile to central spikelets but are developmentally delayed. The inflorescence base was specified by a module that included genes such as *VRT2*, *SVP1* (also referred to as *MADS22*), and *TFL1*, which restrict the vegetative-to-reproductive transition and associate with rudimentary spikelet formation at the inflorescence base (*14*–*16*, *18*, *21*, *23*). These results suggest that central spikelets are more fertile because they are more developmentally advanced than apical spikelets at the LP stage, while the delayed development of basal spikelets appears to result from the expression of *SVP* and *TFL* genes in adjacent cells.

In addition to genes expressed in spikelet-associated regions, transcripts for other known regulators of inflorescence architecture localized to the central vasculature of the rachis and inflorescence base. Curiously, these regulators—including *Ppd-1*, *FT2*, and *PDB1*—are known to control spikelet number rather than their configuration, indicating that genes expressed in the vasculature affect the rachis node number (Fig. 6M) (*3*, *5*, *6*, *59*, *72*, *73*). This model is supported by the enrichment of transcripts encoding photosynthesis-related proteins in the rachis vasculature, as reduced chlorophyll accumulation caused by the absence of the vascular-expressed *HvCMF4* (*CCT MOTIF FAMILY4*) restricted the final spikelet number and grain production in barley (*74*). In support of this model, we observed that transcripts encoding photosynthesis-related proteins accumulated in the rachis during the LP stage, which is when rachis greening initiates and genetic differences in spikelet number are determined [e.g., *Ppd-1* near-isogenic lines (NILs) (*6*)]. Rachis-associated cells also showed strong expression of transporter-encoding transcripts, including aquaporins and *PIN1*, at both the DR and LP stages. This observation suggests that assimilate and hormone transport plays a crucial role in early inflorescence development and that these transporter genes could be promising targets for increasing the spikelet number or fertility.

A third major gene expression domain identified in our data corresponds to meristematic cells, characterized by the expression of histones, ribosomes, and known meristem regulators such as *FCP1* and *CLV1* (*33*–*37*). Meristem-associated clusters were detected in the outer layers of both apical and lateral regions of the developing inflorescence, consistent with the localized expression patterns of key meristem regulators in maize and barley. These results indicate that similar cell types contribute to spikelet development along its entire length, rather than originating from a single pool of cells at the apex. These clusters consistently expressed histone- and ribosome-encoding genes at both the DR and LP stages, suggesting that meristematic cells are maintained during spikelet differentiation, potentially to support the ongoing development of florets and floral organs. The substantial presence of these clusters at the LP stage indicates that depletion of meristematic cells is not the cause of spikelet development terminating; nonetheless, on the basis of evidence from barley and maize, the spikelet or floret number could be boosted in wheat by increasing the activity of meristem maintenance genes (*25*, *35*, *37*).

In summary, these data provide a valuable resource for identifying genes that regulate inflorescence development in wheat, which would be difficult to obtain using traditional genetic approaches, given the complexity and redundancy of the hexaploid genome. Together with functional gene characterization, our findings suggest that genes expressed in the spikelet primordium and boundary regions act cooperatively to shape spikelet architecture, while transcripts enriched in the rachis and inflorescence base influence the spikelet number and fertility. Given that genetic variation underlying yield-related traits has been studied extensively in wheat, we propose that the integration of our spatial transcriptome data with identified quantitative trait loci and genome sequence information will offer a powerful approach to identify candidate genes associated with grain production (*5*, *11*, *75*). In this context, a key advantage of hexaploid wheat is that the expression of all three homeologs in a specific region can serve as a strong filter for identifying candidate genes—an approach supported by our identification of *RA2*, which, in addition to its developmental role, confers a yield advantage when the mutant allele introgressed into elite cultivars (fig. S18). Together with complementary but technically distinct studies, these data provide a valuable framework for deciphering the genes and biological processes that govern the spikelet number and arrangement in wheat inflorescences (*21*–*24*). When combined with similar studies in maize, barley, and rice, this work may help uncover the genetic basis of inflorescence architecture diversity among our major cereals (*20*, *21*, *37*).

## MATERIALS AND METHODS

### Plant materials and growth conditions

The bread wheat (*T. aestivum*) cultivar Mace (Australian Grain Technologies) was used for the spatial transcriptome analysis; Mace is a photoperiod insensitive spring cultivar whose flowering behavior is conferred by the *Ppd-D1a* and *Vrn-B1a* alleles. The *ps3* and *CAD0289* mutant lines used in this study were identified from a screen of the hexaploid wheat ethyl methanesulfonate-induced TILLING population (*cv.* Cadenza), as described previously. The *ps3* NILs used for the phenotype and RNA-seq analyses were derived from *CAD1591*, crossed to *cv.* Cadenza to generate BC_3_F_4-5_ lines that segregated either for the WT or mutant allele of *RA-D2*; the WT genotype was the NIL derived from segregating populations that did not form paired spikelets. A similar approach was used for *CAD0289*, except that phenotype analysis was performed using BC_2_F_2-3_ generation. For both *ps3* and *CAD0289*, we analyzed two independent families generated from separate crosses to *cv.* Cadenza. The elite lines containing the *ra-D2* (A91T) allele were generated by crossing BC_2_F_4_ lines from *CAD1591* × Cadenza families to *cv.* Mace, *cv.* Rockstar (Intergrain), and *cv.* Sheriff CL Plus (Intergrain). Marker-assisted selection of heterozygous individuals was used in a backcrossing program with the elite lines as recurrent parents to generate BC_3_F_4_ lines containing either the mutant or WT *RA-D2* alleles, which were used to analyze yield-component traits.

Plants were grown in glasshouses under long-day (16-hour light/8-hour dark) photoperiods with Heliospectra light-emitting diode lights (Heliospectra, Sweden), with day and night temperatures of 20° and 15°C, respectively. Extralong daylength conditions (to 22-hour light/2-hour dark) were used for the accelerated generation of backcrossed germplasm for *CAD1591* and *CAD0289*. Glasshouse-grown plants used for phenotypic analyses were grown in coco peat soil mix in 3.7-liter pots, with five replicates sown per genotype in a randomized design. The field trial was conducted at Roseworthy, South Australia (34°31′37.9″S, 138°41′21.0″E). The site contains loam over red clay soils, and the trial was sown in early May 2023, with nitrogen applied at 100 kg/ha across two intervals (seeding and early tillering). The field trial was designed in a randomized complete block design, with three replicate blocks.

### Stereo-seq capturing and library construction

The library preparation and sequencing process for Stereo-seq was based on the modified version of the Stereo-seq standard protocol (version 1.1). Embedded tissues were sectioned longitudinally at a thickness of 12 μm using a Leica CM1800 cryostat. The tissue sections were adhered to the surface of the Stereo-seq chip and incubated at 37°C for 2 min. They were then fixed in methanol and incubated at −20°C for 30 min. Subsequently, the sections were stained with Qubit ssDNA stain to label nuclei and Fluorescent Brightener 28 (Sigma-Aldrich, F3543-5g) for cell wall visualization (fig. S25). Tissue integrity and sectioning quality were evaluated by image-based quality control under fluorescence microscopy before proceeding. The sections were then decross-linked in TE buffer (10 mM tris and 1 mM EDTA, pH 8.0) at 55°C for 1 hour. Afterward, the sections were permeabilized at 37°C for 12 min to enable efficient mRNA capture by spatially barcoded probes, followed by on-slide cDNA synthesis, and incubated overnight at 42°C for reverse transcription and cDNA synthesis. The tissue was subsequently digested at 37°C for 30 min and treated with exonuclease I (NEB, M0293L) for 1 hour to remove free nucleic acids. Resulting cDNA libraries were quantified using Qubit and assessed for size distribution using TapeStation. Last, pooled libraries were sequenced on the MGI DNBSEQ-G400 platform [with either 50– or 100–base pair (bp) paired-end sequencing].

### Stereo-seq data processing and quality control

Stereo-seq raw data processing and quality control were performed using the SAW software suite (*76*). Read 1 contained the coordinate identity (CID) and molecular identifier (MID) sequences, with CID spanning nucleotides 1 to 25 and MID spanning nucleotides 26 to 35. Read 2 comprised the cDNA sequence. During the initial processing, CID sequences were mapped to the predefined coordinates of the in situ captured chip, allowing for a single-base mismatch to account for sequencing and polymerase chain reaction (PCR) errors. Reads with MID sequences containing ambiguous “*N*” bases or more than two bases with quality scores below 10 were excluded. The CID and MID identifiers were appended to the respective read headers.

The retained reads were then aligned to reference genomes (IWGSC RefSeq version 1.1) using the STAR aligner. Only mapped reads with a mapping quality score greater than 10 were annotated to their corresponding genes. Unique molecular identifiers (UMIs) with identical CIDs and gene loci were merged, allowing for a single-base mismatch to correct sequencing and PCR errors. The single-stranded DNA photo feature in the SAW workflow enabled accurate mapping of raw reads to their precise positions on the tissue. Last, an expression count matrix was generated, documenting the expression levels of each gene in every spot.

To verify the gene expression profiles mapped to the DR and LP inflorescences, we compared localized transcript signals from our dataset to those identified using MERFISH (*21*). This comparison included 20 and 23 selected genes from DR and LP, respectively, that displayed representative expression of the identified clusters and was complemented by analysis of 20 random genes from the MERFISH panel. We also compared our data to reported spatial expression patterns for genes analyzed using in situ hybridization, including *ALOG1* (*3*), *LAX1* (*77*), and *VRT2* (*18*). Spatial gene expression values for the DR and LP stages were log normalized and compared to corresponding bulk RNA-seq samples (*3*) using the 30,000 most highly expressed genes. Gene-wise concordance between bulk and spatial datasets was assessed using a Spearman correlation in R Studio.

### Unsupervised clustering analysis of Stereo-seq data and cluster comparisons

We performed unsupervised clustering analysis using the Seurat R package (version 4.4.3). First, we normalized the UMI count matrices using the SCTransform function. Feature genes were then selected with the “FindVariableGenes” function using the vst method and retaining 2000 variable features. Subsequently, we conducted principal components analysis (PCA) using the RunPCA function on the selected variable genes, retaining 30 principal components for dimensionality reduction. Next, we constructed the shared nearest-neighbor graph using the FindNeighbors function and performed clustering with the Louvain algorithm via the FindClusters function. The clustering resolution parameters were adjusted according to the specific datasets. For visualization, we used nonlinear dimensionality reduction algorithms (RunUMAP). Batch effects between samples were corrected using the “RunHarmony” function to ensure that observed differences in gene expression are due to biological rather than technical variations.

To quantify transcriptional similarity across cellular identities, we calculated the mean expression profiles per cell population using “AverageExpression” in Seurat. These aggregated expression vectors were subsequently analyzed via Pearson correlation to assess relationships between distinct cell clusters. The pairwise correlation heatmap was plotted in R with the function of pheatmap.

To investigate the conservation of cell-identify specific genes during inflorescence development at two stages, we used scanpy and plotly packages (in Python) to construct Sankey diagrams visualizing the dynamic mapping of cell type–specific marker genes between the two developmental stages. The width of each flow reflects the relative conservation or transition of marker gene signatures across time points, with distinct colors representing different cell lineages.

### Identification of marker and cluster-enriched genes

To identify cluster-enriched genes within the spatial transcriptome data, we used the FindAllMarkers function in Seurat. We applied a two-sided Wilcoxon rank-sum test to compare gene expression levels between clusters, setting a log fold change threshold of 1.5 (logfc.threshold = 0.58) to highlight genes with at least a 1.5-fold expression difference. We also required that genes be expressed in at least 25% of the cells within a given cluster (min.pct = 0.25) to focus on biologically relevant markers. Last, we controlled for multiple comparisons using the Bonferroni correction, retaining only genes with an adjusted *P* value below 0.05—these are the marker genes for each cluster (table S2). These marker genes were used to generate the Venn diagrams and the Sankey plots that compare expressed genes from each cluster across the two developmental stages (Fig. 2). Enriched genes for each cluster were identified from the complete set of expressed genes as those whose expression within a given cluster accounted for at least 30% of the total expression of that gene across all clusters and whose total expression score was above a given threshold (0.2 to 0.8, depending on cluster grouping) (table S3).

### Monocle2 analysis

To investigate the developmental trajectory of cell clusters in the DR and LP tissues, we used Monocle2 (*57*). Specifically, we extracted data from the key clusters: DR_C1, DR_C2, DR_C3, DR_C5, DR_C6, DR_C7, DR_C8, and DR_C10 and LP_C2, LP_C3, LP_C4, LP_C5, LP_C6, LP_C7, LP_C9, LP_C10, and LP_C11. To identify the most informative genes for this analysis, we used the “FindAllMarkers” function from Seurat (version 4.4.3) to calculate the top marker genes for each cluster. These marker genes were then selected for subsequent analysis with Monocle2. Dimensionality reduction was performed using the DDRTree method, which helped to reconstruct the developmental trajectory. The resulting trajectory was visualized using the plot_cell_trajectory function. In this analysis, the cluster corresponding to the shoot apical meristem (DR_C2 and LP_C3) was designated as the starting point. In addition, we identified a branch point to analyze divergent differentiation pathways. We applied the BEAM (branch expression analysis modeling) algorithm to detect genes that are differentially expressed in a pseudotime-dependent or branch-dependent manner. The number of gene expression profiles was defined using the num_clusters parameter. Inspection of the inferred pseudotime trajectory for DR and LP indicated three major cell states, together with minor transitional states; therefore, genes were initially partitioned into four expression profiles to capture these dynamics. Genes that exhibited significant branch dependency and were assigned to these profiles were visualized using the plot_genes_branched_heatmap function. This approach allowed us to elucidate the dynamic gene expression changes underlying the developmental progression of the early wheat inflorescence.

### Spatial coexpression module analysis

To identify gene coexpression modules in spatially resolved transcriptomic data, the Hotspot algorithm (version 1.1.1) was used. The spatial transcriptomic data were first converted into a Hotspot object using the Hotspot function. A *K*-nearest neighbors graph was then constructed using the create_knn_graph function with n_neighbors set to 300 to capture the spatial relationships between cells. Genes were grouped into coexpression modules using the “create_modules” function, with a minimum gene threshold of 15 and a false discovery rate threshold of 0.05 to ensure the detection of biologically meaningful and statistically significant modules. Last, the “calculate_module_scores” function was used to compute module scores for each cell, identifying cells that highly express the genes within specific modules.

### GO enrichment analysis

To further elucidate the biological functions of the cluster-enriched genes, we performed Gene Ontology (GO) enrichment analysis using the clusterProfiler package (version 4.12.6). This analysis identified overrepresented biological processes, molecular functions, and cellular components among the cluster-enriched genes. The results of the GO enrichment analysis were visualized using the ggplot2 package (R Studio).

### Fine mapping of the *ra-D2* allele in *ps3* mutant lines

To fine-map the causal mutation in *ps3*, two independent segregating BC_2_F_2_ families were generated from a cross between *CAD1591* and *cv.* Cadenza. Two bulk DNA samples were generated for each of the two families: The WT bulk included DNA of individuals that produced normal inflorescences without paired spikelets, and the mutant bulk included DNA of individuals that produced paired spikelets (more than eight secondary spikelets per inflorescence). Genomic DNA was extracted from leaves, as described previously (*11*). Exome capture sequence analysis was performed as described previously (*11*), with identified single-nucleotide polymorphisms (SNPs) compared between the two families to eliminate noncausal variant alleles, and SNPs were verified by cross-referencing the identified alleles to those identified previously for *CAD1591* (*78*). Loci on chromosomes 3D and 6A were identified as potential regions for the causal mutation.

To refine the mutation site, paired spikelet–producing BC_2_F_3_ plants were crossed to Cadenza and self-fertilized to generate BC_3_F_2_ families. Individual lines were phenotyped for paired spikelets and genotyped using Kompetitive allele–specific PCR–based markers for the SNPs identified using exome capture analysis. This analysis identified a region between chr3D.39387558-76931611 as the unique locus associated with paired spikelet production. Individuals that displayed heterozygosity within the region on chromosome 3D were used to generate homozygous recombinant inbred lines with different segments of the identified locus. Subsequent phenotype analysis of lines containing either variant or reference alleles for each site across the locus defined marker chr3D.47494317 (G>A; *TraesCS3D02G093500*) as a unique allele associated with paired spikelet production in all lines (6 to 12 replicate plants per line). The statistical difference in phenotype data for the recombinant inbred lines, relative to the WT, was performed using Dunnett’s test. The sequence of *TraesCS3D02G093500* was examined in WT and mutant NILs using genomic DNA and complementary DNA, which confirmed a G>A mutation at 271 bp of the coding region for *TraesCS3D02G093500*. A similar crossing strategy with marker-assisted selection was used for the analysis of BC_2_F_2_ plants derived from the cross between *CAD289* and Cadenza, with phenotype analysis performed on individuals identified to be homozygous for either the WT or mutant allele, using the marker chr3D.47493729. Oligonucleotide sequences for Kompetitive allele–specific PCR markers are provided in table S10.

### Phenotypic analyses

Rachis nodes (primary spikelets) and secondary spikelets were recorded for inflorescences of the main stem and first tiller, as described previously (*5*). Secondary spikelet distribution was determined as described previously (*5*) using inflorescences from the main stem. The rate of inflorescence development was determined for the *ps3* and *CAD0289* mutant lines, relative to their respective WT siblings, by measuring the inflorescence length at intervals defined by leaf emergence (leaves 4 to 7). The flowering time was determined for each genotype at the emergence of the inflorescence from the boot. Scanning electron microscopy was performed on developing inflorescences of WT and *ps3* mutants at the late TS stage, as described previously (*11*). Replicate numbers for each phenotype analysis are provided in figure legends.

For glasshouse grown plants in the *cv.* Mace and *cv.* Rockstar backgrounds, we measured the total grain number and grain weight per plant (five plants) and the secondary spikelet number for two spikes per plant (10 spikes). For the field grown plants, the grain number, grain weight, and secondary spikelet number per spike were measured for three random spikes harvested from each of the three replicate blocks (nine spikes in total). Statistical differences in phenotype data for lines carrying the mutant alleles for *RA-D2* (Cadenza and elite types), relative to their WT siblings, were determined using a two-sided Student’s *t* test.

### RNA extractions and RNA-seq transcriptome analysis

RNA was extracted from whole inflorescences at the DR, LP, and TS stages of early inflorescence development. Each sample consisted of a pooled collection of 6 to 12 inflorescences, stored in a DNA/RNA Shield solution (Zymo Research, R1100-50) at −20°C. Three biological replicates were collected for *ps3* and the WT sibling line for each developmental stage. Total RNA was extracted using TRIzol/chloroform (TRI Reagent, Sigma-Aldrich; chloroform, ≥99.8%; Thermo Fisher Scientific, US) and purified using the RNA Clean and Concentrator kit (Zymo Research, R1013) following the protocol outlined by Millán Blánquez *et al.* (*79*). The RNA concentration was measured using the Invitrogen Qubit 3 Fluorometer and Qubit RNA HS (High Sensitivity) Assay Kit (Invitrogen, Thermo Fisher Scientific, US), and RNA integrity was examined using the Agilent 5400 Fragment Analyzer (Agilent Technologies, US). Library construction and RNA-seq were performed by Novogene (Novogene HK Company Ltd., Hong Kong). Sequencing libraries were generated using the NEBNext Ultra RNA Library Prep Kit for Illumina (NEB), and index codes were added to attribute sequences to each sample. For sequencing, clustering of the index-coded samples was performed on the cBot Cluster Generation System using the TruSeq PE Cluster Kit v3-cBot-HS (Illumina, US). After cluster generation, the libraries were sequenced on an Illumina NovaSeq platform to generate 150-bp paired-end reads.

Raw reads were trimmed and filtered using fastp tool (version 0.20.0) (*80*), and cleaned reads were pseudoaligned to IWGSC Chinese Spring gene model index version 1.1 using kallisto (version 0.46.1) (*81*), as described previously (*11*). On average, more than 77% of raw reads were successfully pseudoaligned to the reference transcriptome and used for transcript quantification. Differential gene expression analysis was performed using the R package DESeq2 (version 1.49.3) (*82*) with default parameters. Only transcripts/genes with expression level ≥0.5 transcripts per million (TPM) in one sample at least were included. Transcripts/genes with a false discovery rate–adjusted *P* value (*q* value) <0.05 were considered differentially expressed. These were further categorized into lists of up- and down-regulated genes at the DR, LP, and TS stages in *ps3* for each pairwise comparison, relative to the WT sibling, based on *q* < 0.05 and mean TPM fold change >0.5.
