# Supplementary material for: Spatial transcriptomics identifies distinct domains regulating yield-component traits of the wheat ear
Source: Sci Adv. 2026 Jun 17;12(25):eaed1407. doi: 10.1126/sciadv.aed1407 (PMC13274600; doi:10.1126/sciadv.aed1407)
Supplement: Supplementary file 1 — Figs. S1 to S25 Table S10 Legends for tables S1 to S9 [file sciadv.aed1407_sm.pdf]

Science Advances
AAAS

Supplementary Materials for

Spatial transcriptomics identifies distinct domains regulating yield-component traits of the wheat ear

Yue Qu *et al.*

Corresponding author: Scott A. Boden, scott.boden@adelaide.edu.au

Sci. Adv. **12**, eaed1407 (2026)
DOI: 10.1126/sciadv.aed1407

The PDF file includes:

Figs. S1 to S25
Table S10
Legends for tables S1 to S9

Other Supplementary Material for this manuscript includes the following:

Tables S1 to S9

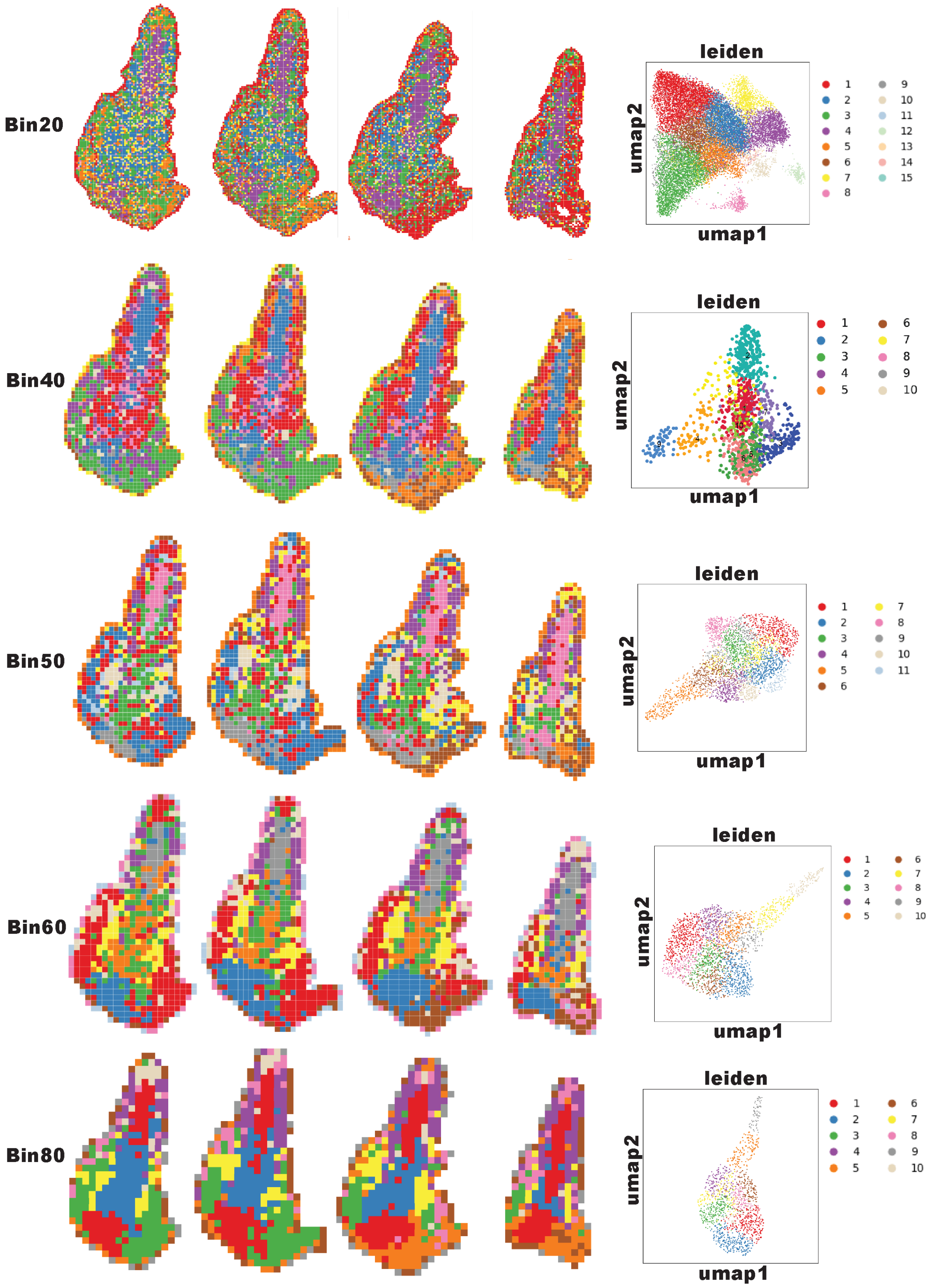

Supplementary Figure 1. Overview of spatial resolution at different bin sizes in double ridge (DR) stage sections. Spatial maps and UMAP projections of DR stage sections showing cluster resolution at bin sizes including Bin20, Bin40, Bin50, Bin60, and Bin80. The Bin40 images are also shown in Fig. 1.

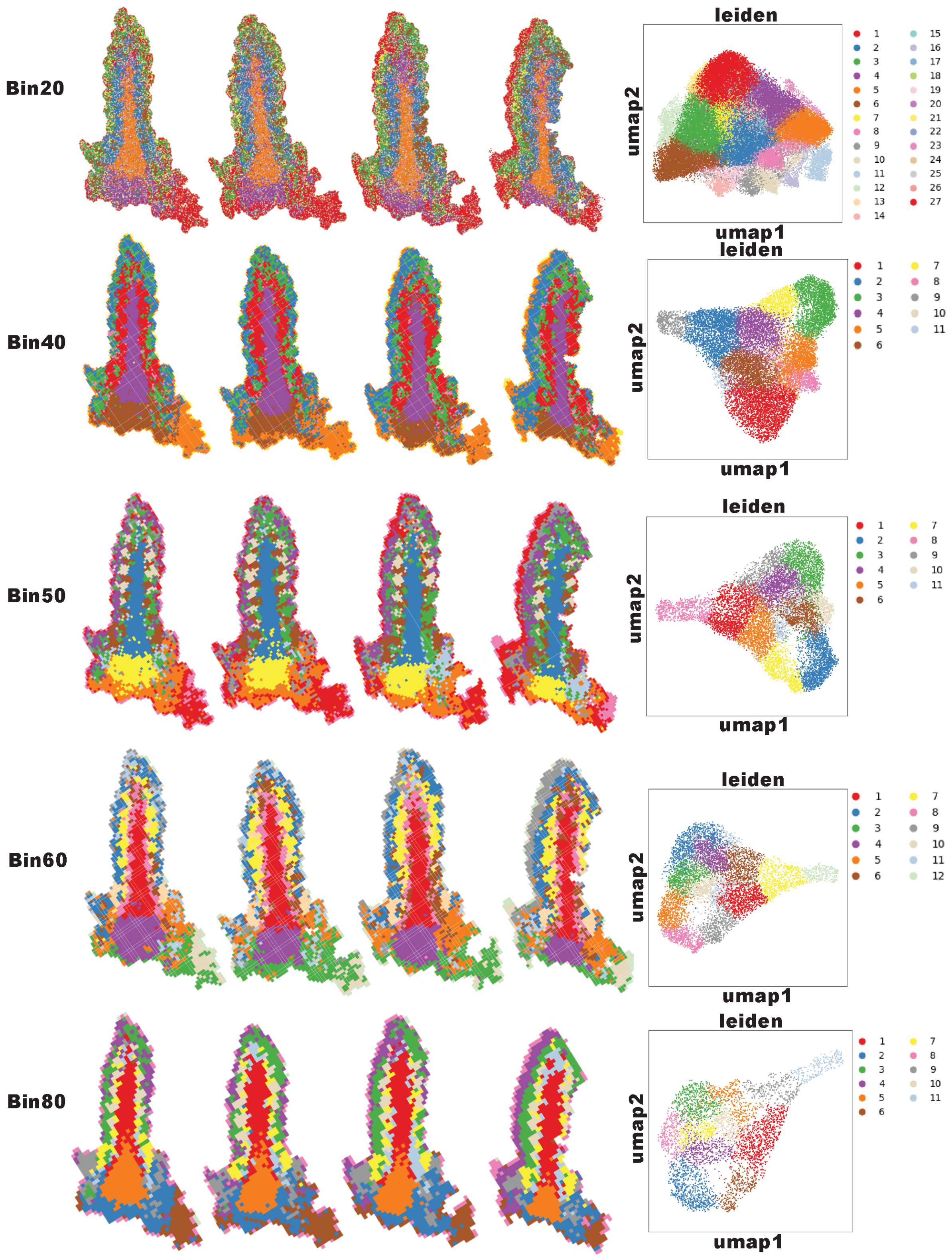

Supplementary Figure 2. Overview of spatial resolution at different bin sizes in lemma primordia (LP) stage sections. Spatial maps and UMAP projections of LP stage sections showing cluster resolution at bin sizes including Bin20, Bin40, Bin50, Bin60, and Bin80. The Bin50 images are also shown in Fig. 1.

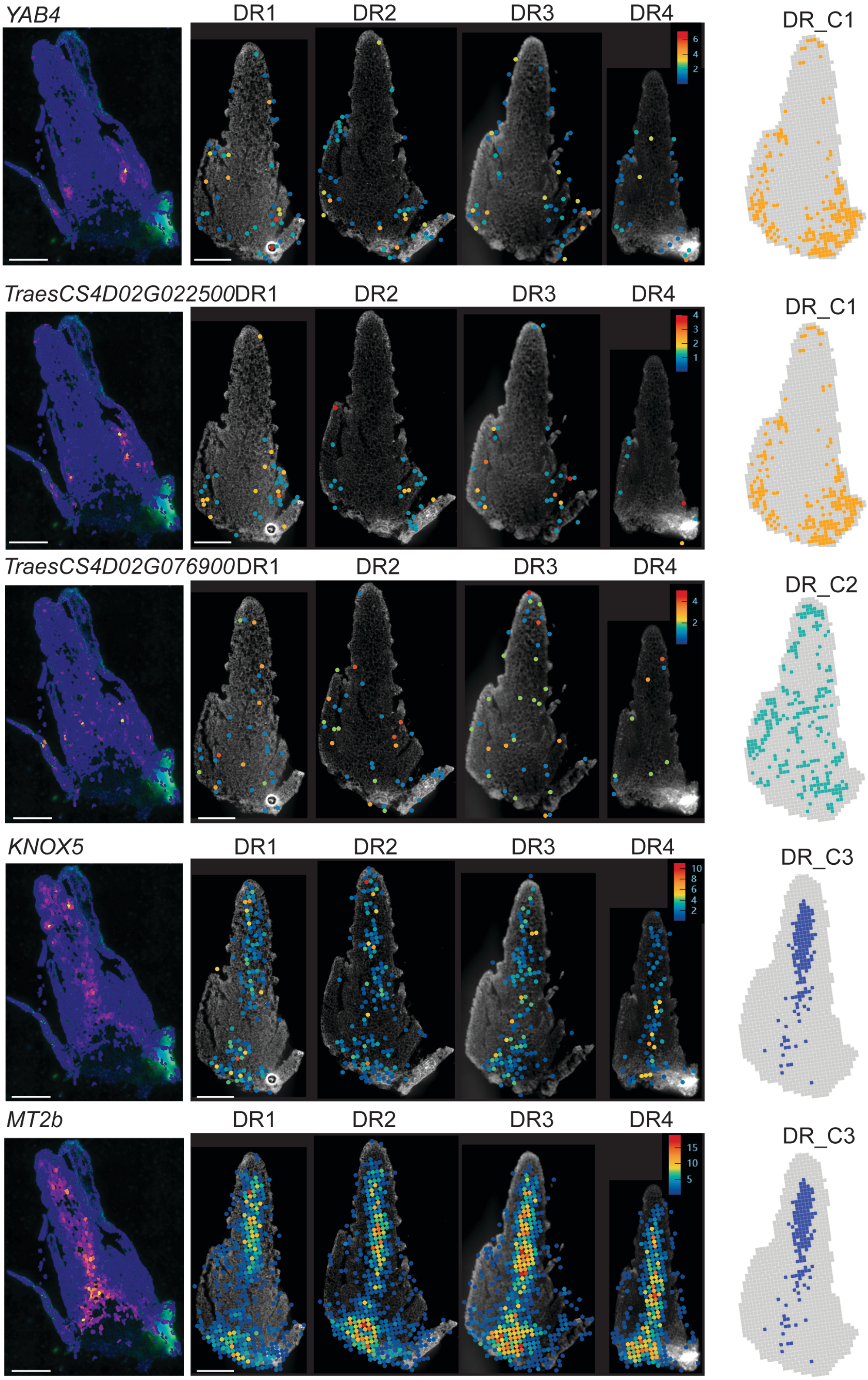

Supplementary Figure 3. Validation of DR_C1 to C3 cluster-enriched genes with MERFISH data. Comparison of gene expression profiles for DR cluster 1 to 3 between spatial transcriptomics and publicly available MERFISH data (*21*) (left), showing consistent spatial localization. Genes including *YAB4*, *TraesCS4D02G022500*, *TraesCS4D02G076900*, *KNOX5*, and *MT2b*. Scale bars, 200 µm.

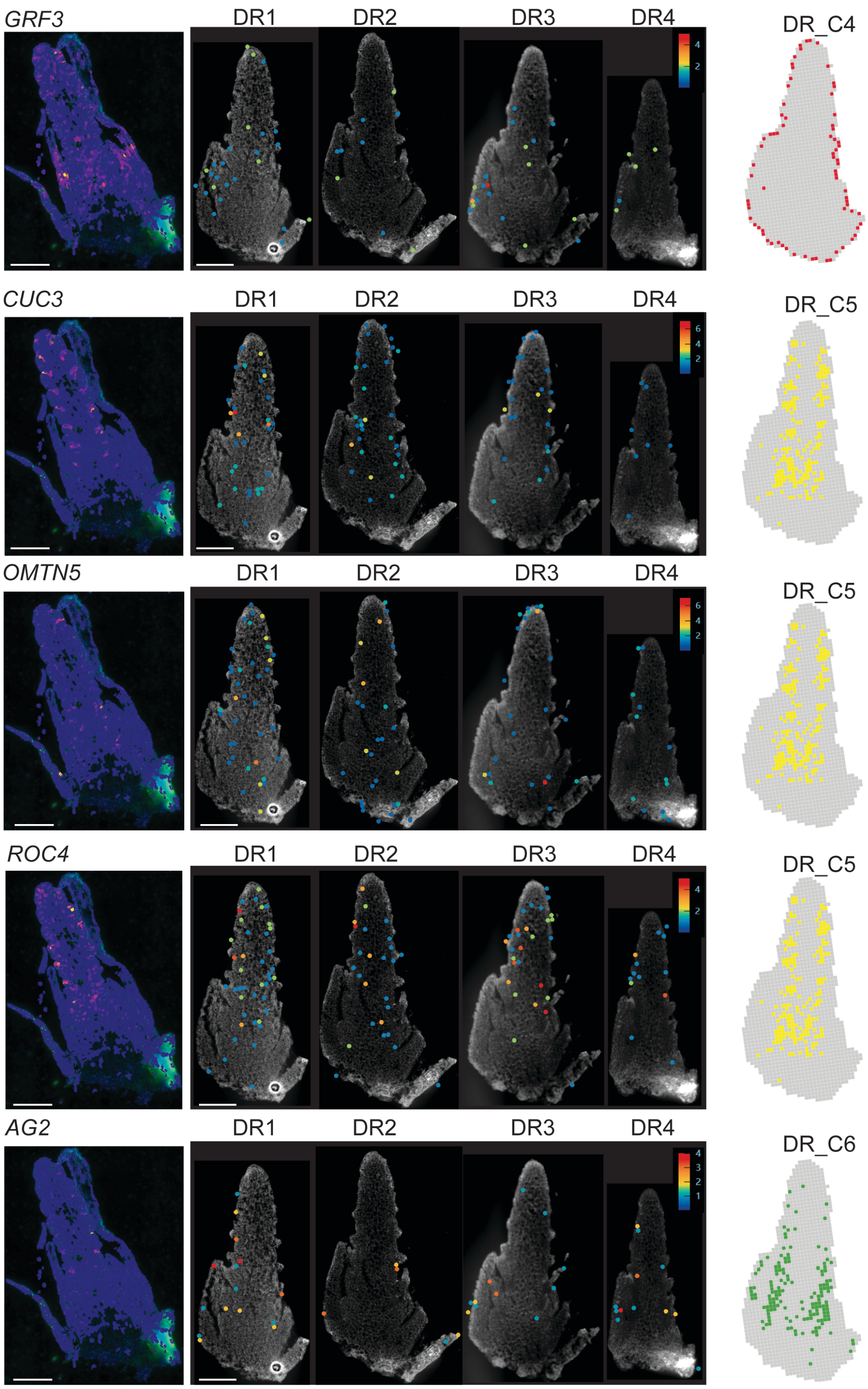

Supplementary Figure 4. Validation of DR_C4 to C6 cluster-enriched with MERFISH data. Comparison of gene expression profiles for DR cluster 4 to 6 between spatial transcriptomics and publicly available MERFISH data (*21*) (left), showing consistent spatial localization. Genes including *GRF3*, *CUC3*, *OMTN5*, *ROC4*, and *AG2*. Scale bars, 200 µm.

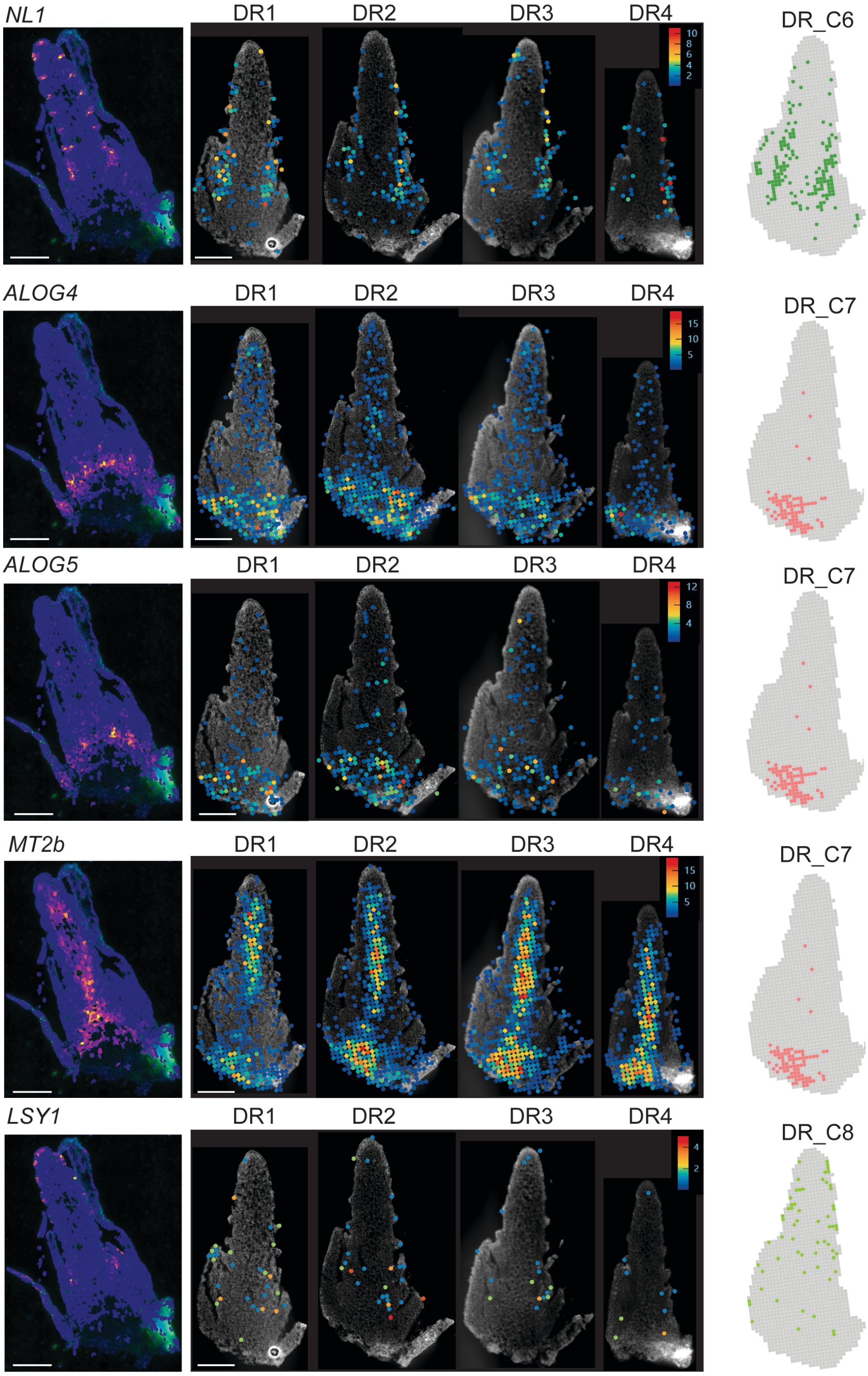

Supplementary Figure 5. Validation of DR_C6 to C8 cluster-enriched with MERFISH data. Comparison of gene expression profiles for DR cluster 6 to 8 between spatial transcriptomics and publicly available MERFISH data (*21*) (left), showing consistent spatial localization. Genes including *NL1*, *ALOG4*, *ALOG5*, *MT2b*, and *LSY1*. Images of *NL1* (Fig. 1) *MT2b* (Fig. 1) and *ALOG4* (Fig. 4) are shown in main figures, alongside expression detected at LP or other genes/homeologues. Scale bars, 200 µm.

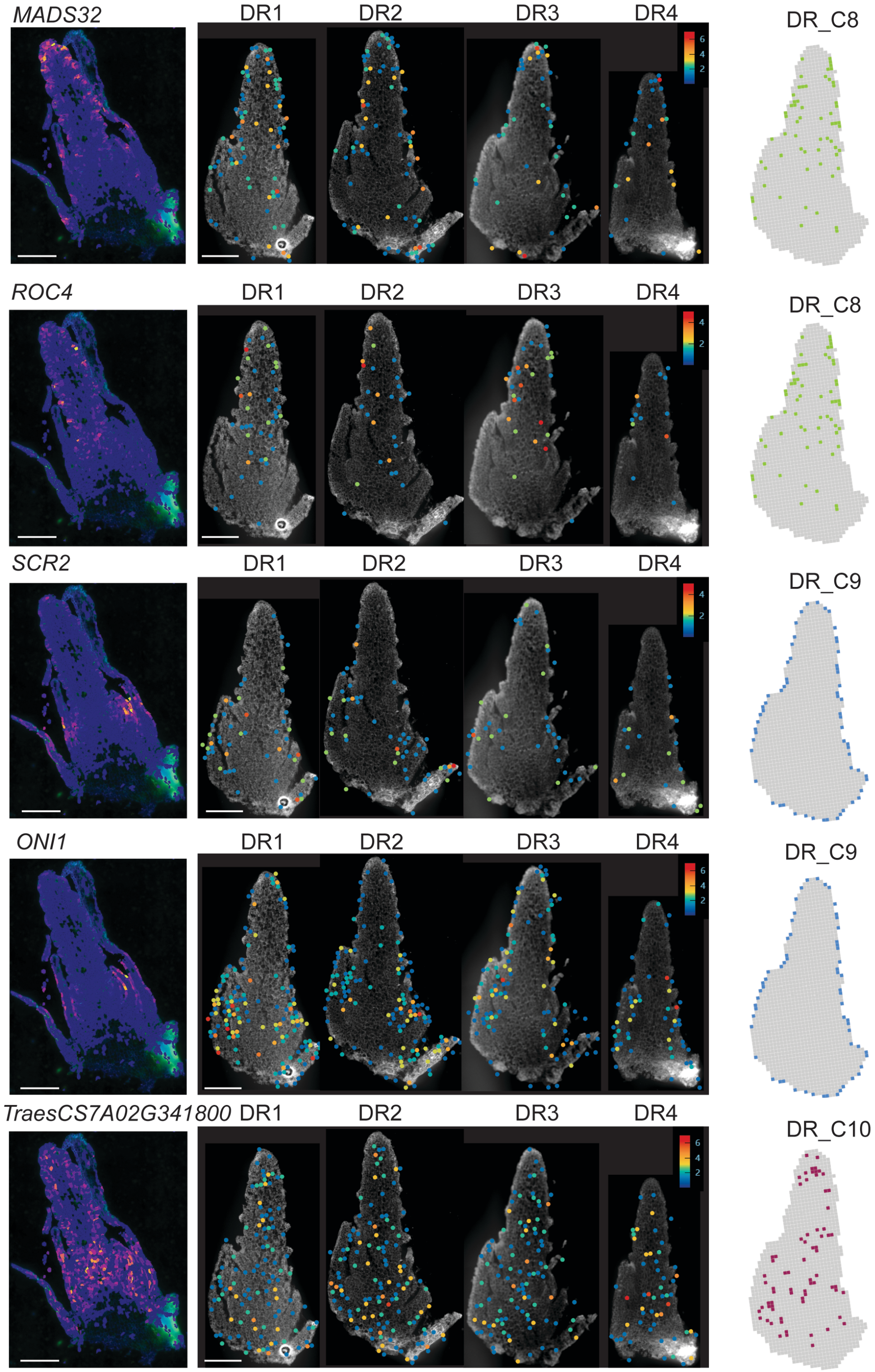

Supplementary Figure 6. Validation of DR_C8 to C10 cluster-enriched with MERFISH data. Comparison of gene expression profiles for DR cluster 8 to 10 between spatial transcriptomics and publicly available MERFISH data (*21*) (left), showing consistent spatial localization. Genes including *MADS32*, *ROC4*, *SCR2*, *ONI1*, and *TraesCS7A02G341800*. Images of *MADS-32* and *ON11* is also shown in Fig. 1, alongside expression detected at LP. Scale bars, 200 μm.

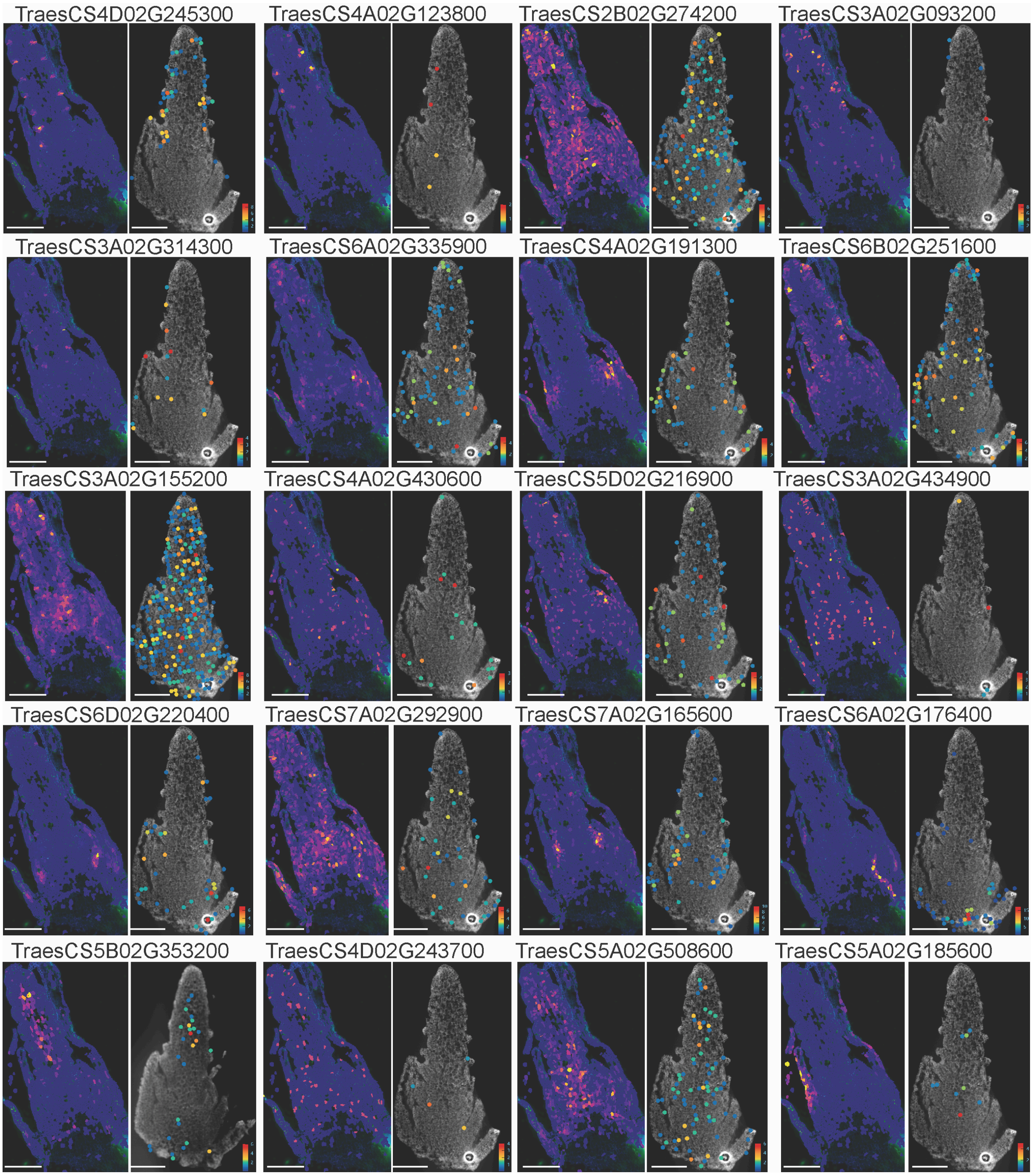

Supplementary Figure 7. Spatial expression comparison of 20 genes randomly selected from the MERFISH panel at the DR stage. Random selection was performed using Excel's RAND() function with fixed values to ensure reproducibility. Stereo-seq maps (right image) show aggregated homeologs expression to match the non–homeolog-specific method of MERFISH (*21*) (left image).

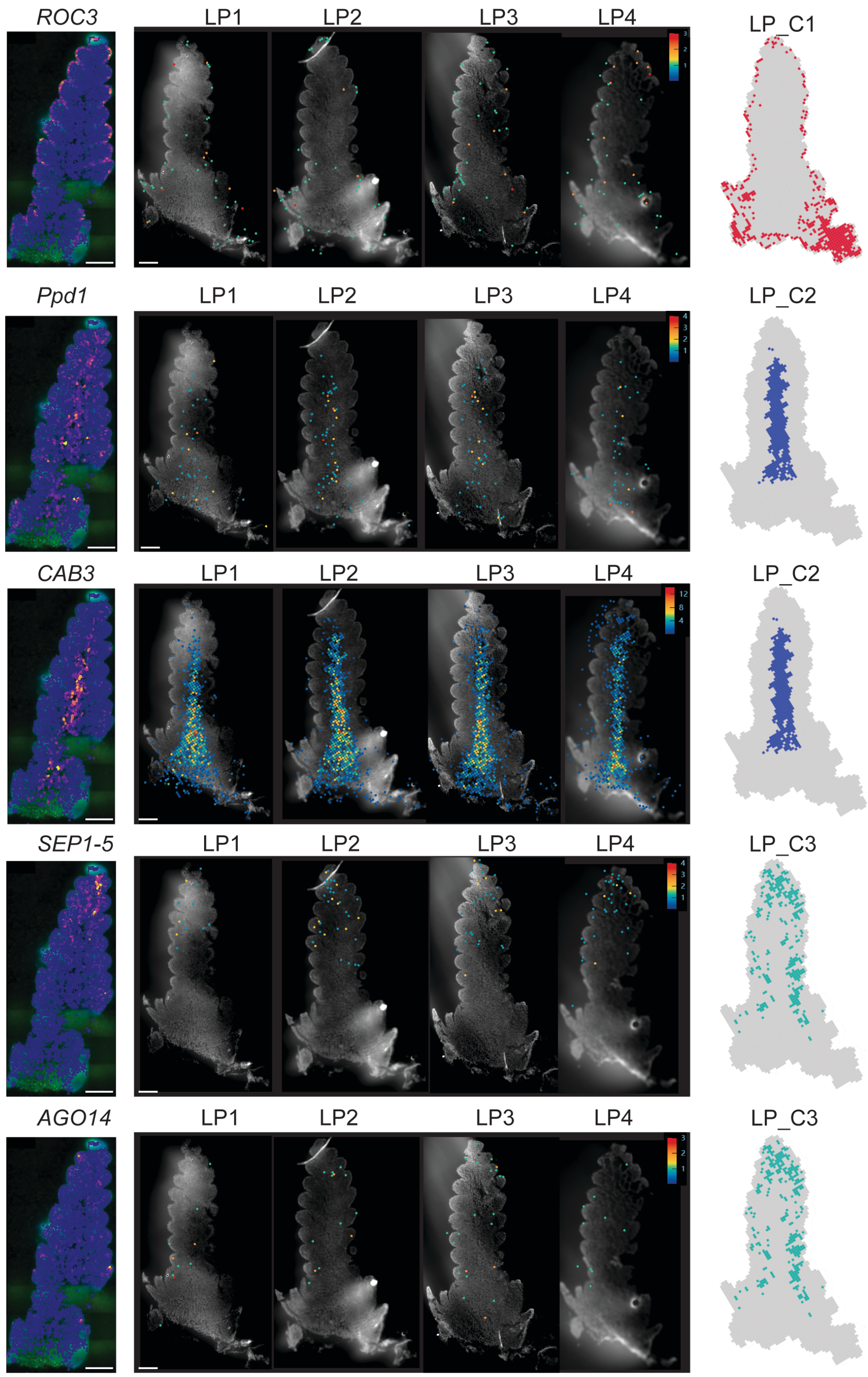

Supplementary Figure 8. Validation of LP_C1 to C3 cluster-enriched with MERFISH data. Comparison of gene expression profiles for LP cluster 1 to 3 between spatial transcriptomics and publicly available MERFISH data (*21*) (left), showing consistent spatial localization. Genes including *ROC3*, *Ppd-1*, *CAB3*, *SEP1-5*, and *AGO14*. An image of *Ppd-1* is also shown in Fig. 4, alongside expression detected at LP. Scale bars, 200 µm.

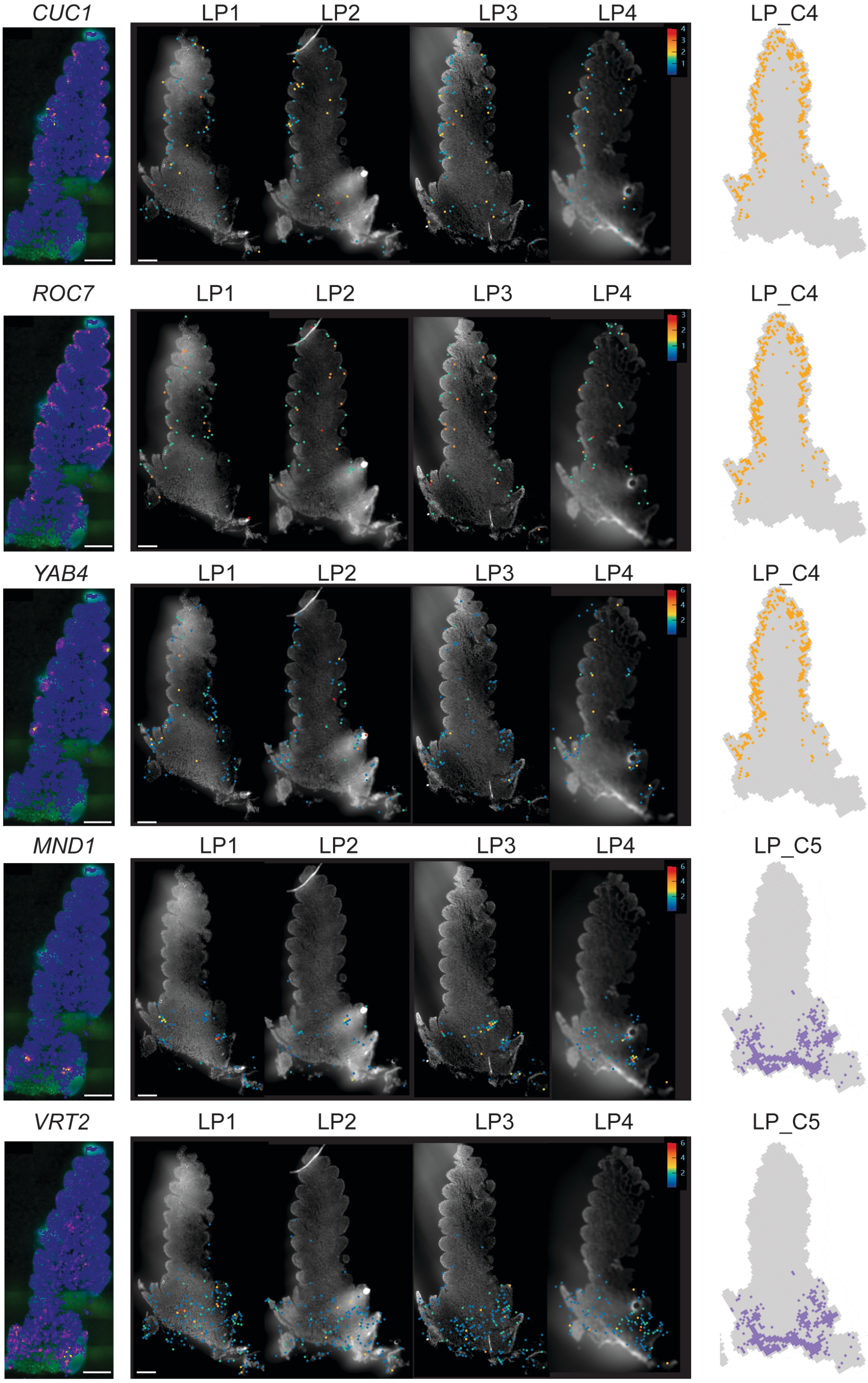

Supplementary Figure 9. Validation of LP_C4 and C5 cluster-enriched with MERFISH data. Comparison of gene expression profiles for LP cluster 4 and 5 between spatial transcriptomics and publicly available MERFISH data (*21*) (left), showing consistent spatial localization. Genes including *CUC1*, *ROC7*, *YAB4*, *MND1*, and *VRT2*. An image of *MND1* is also shown in Fig. 1, alongside expression detected at DR. Scale bars, 200 µm.

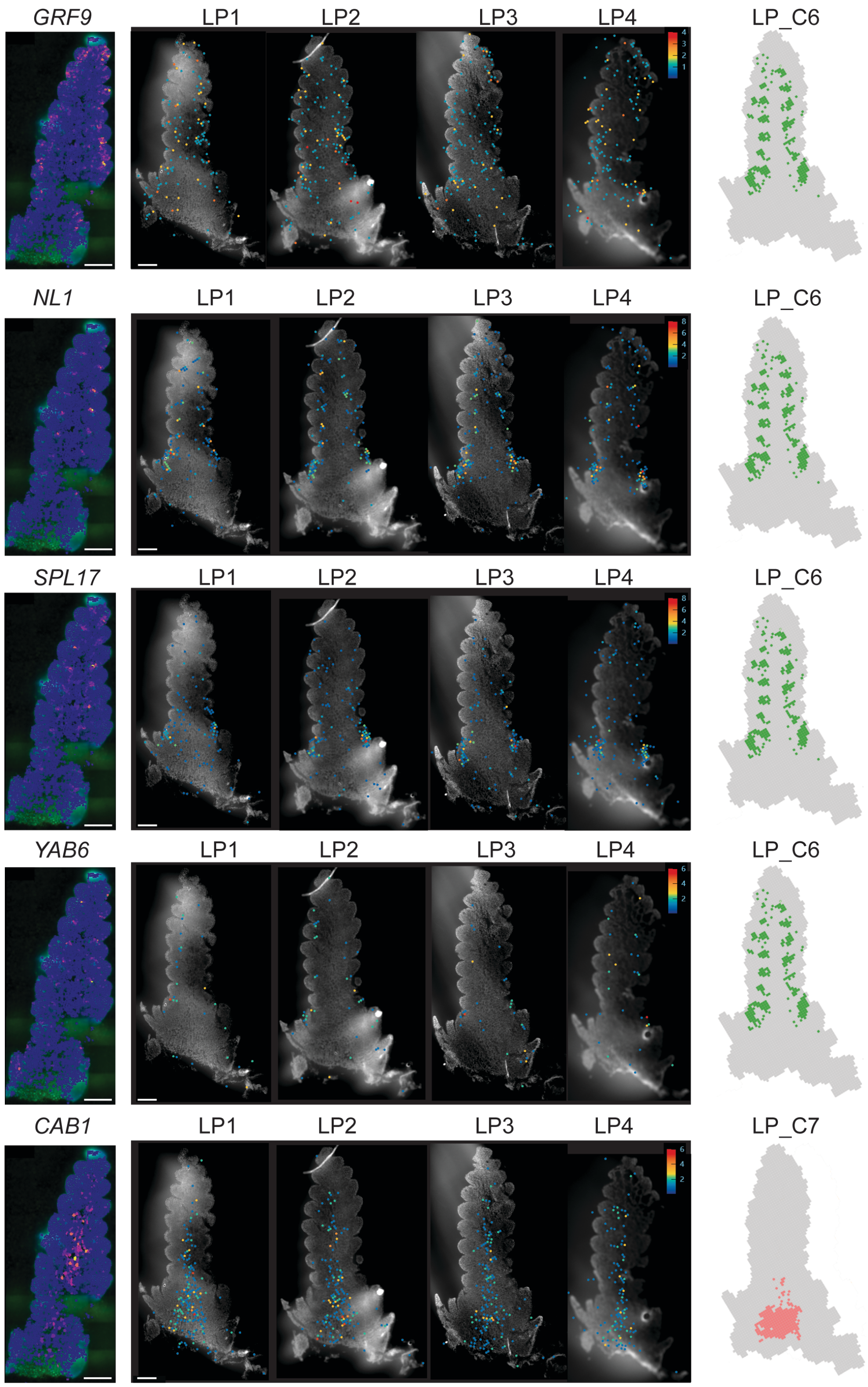

Supplementary Figure 10. Validation of LP_C6 and C7 cluster-enriched with MERFISH data. Comparison of gene expression profiles for LP cluster 6 and 7 between spatial transcriptomics and publicly available MERFISH data (*21*) (left), showing consistent spatial localization. Genes including *GRF9*, *NL1*, *SPL17*, *YAB6*, and *CAB1*. An image of *NL1* is also shown in Fig. 1, alongside expression detected at DR. Scale bars, 200 µm.

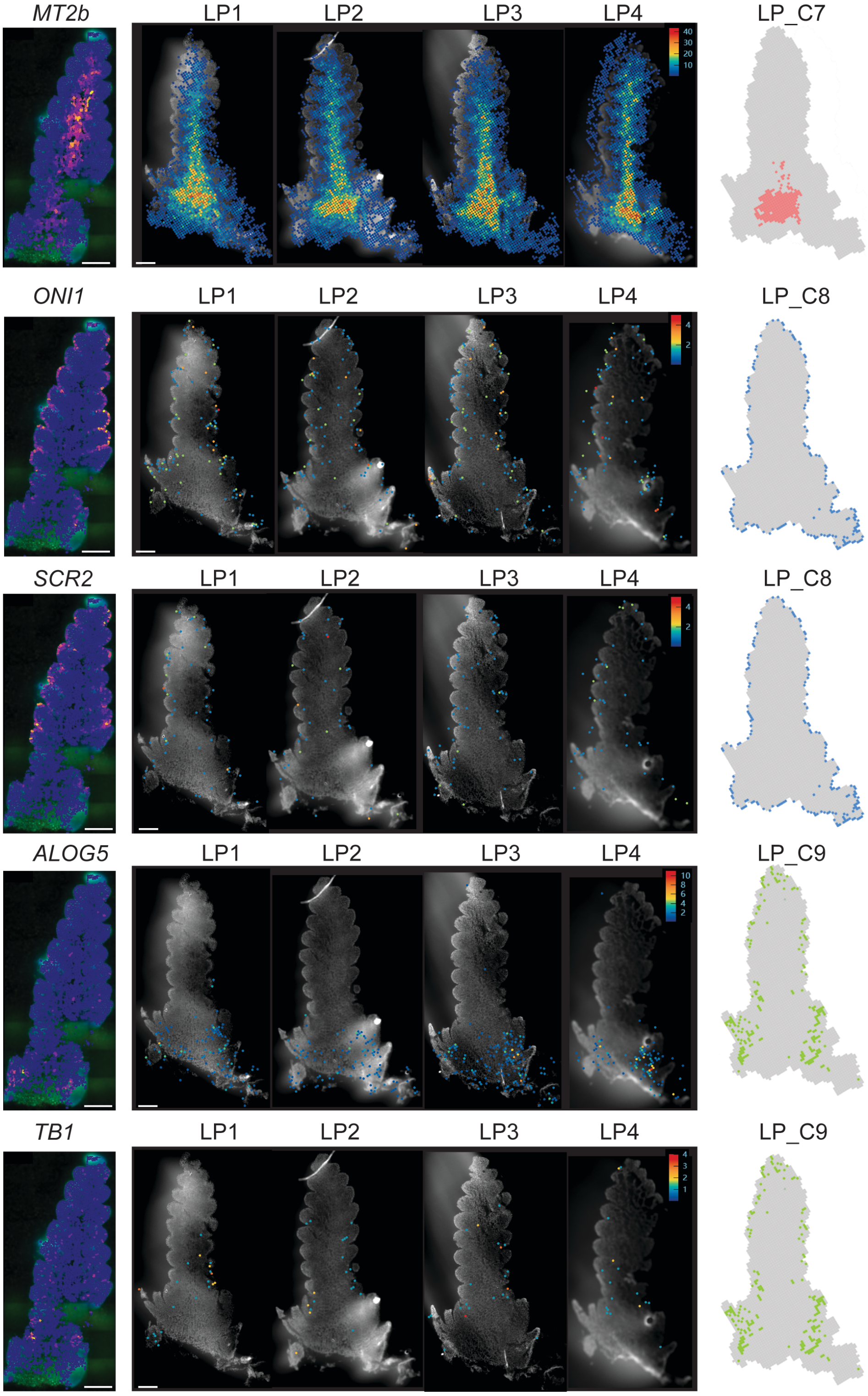

Supplementary Figure 11. Validation of LP_C7 to C9 cluster-enriched with MERFISH data. Comparison of gene expression profiles for LP cluster 7 to 9 between spatial transcriptomics and publicly available MERFISH data (*21*) (left), showing consistent spatial localization. Genes including *MT2b*, *ONI1*, *SCR2*, *ALOG5*, and *TB1*. Images of *MT2b*, *ONI1* and *ALOG5* are shown in Figs. 1, 4 and 5, respectively alongside expression at LP or other genes/homeologues. Scale bars, 200 µm.

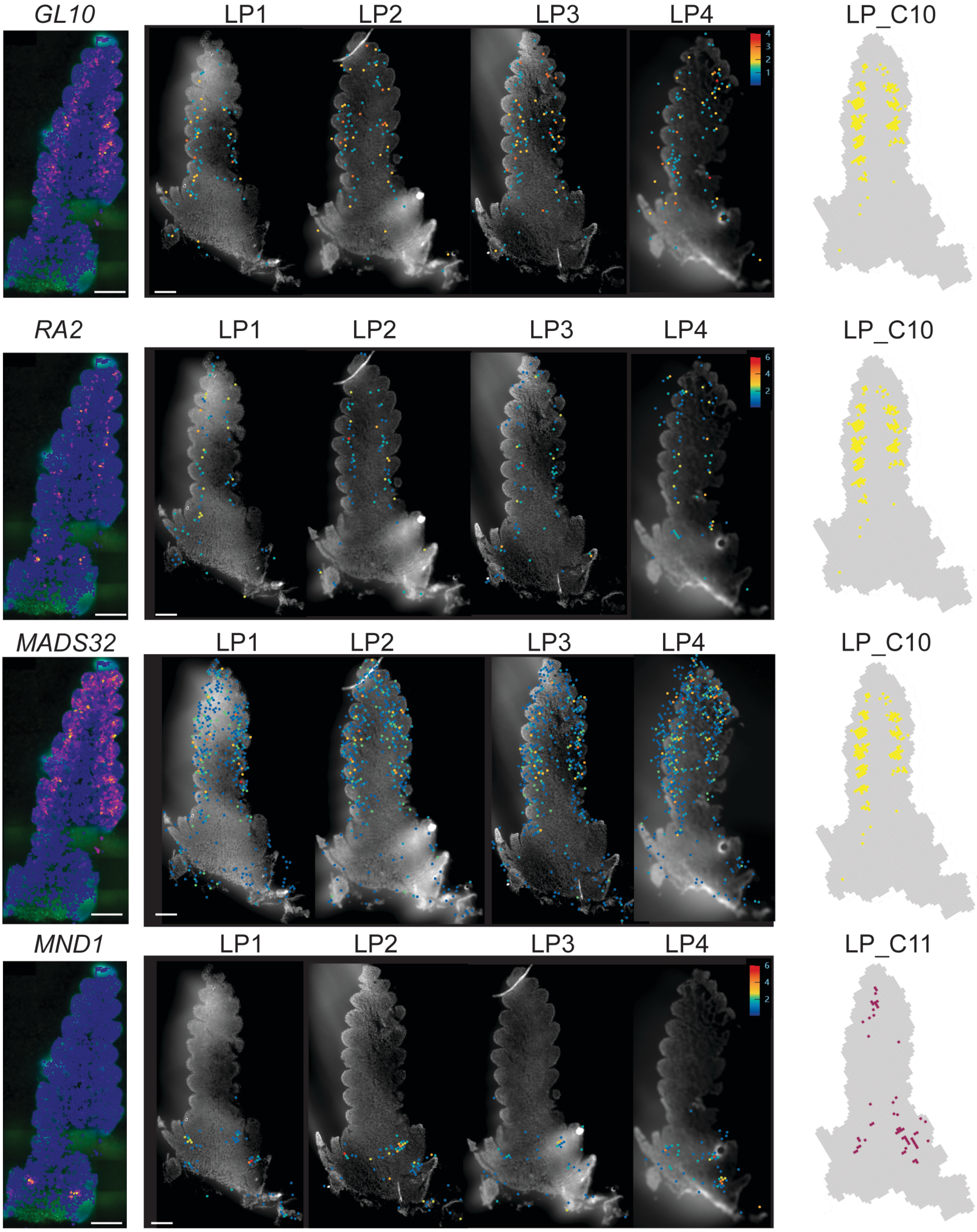

Supplementary Figure 12. Validation of LP_C10 and C11 cluster-enriched with MERFISH data. Comparison of gene expression profiles for LP cluster 10 and 11 between spatial transcriptomics and publicly available MERFISH data (*21*) (left), showing consistent spatial localization. Genes including *GL10*, *RA2*, *MADS32*, and *MND1*. Images of *MND1*, *MADS-32* and *RA-2* are shown in Figs. 1 and 6 alongside expression at DR or other genes/homeologues. Scale bars, 200 µm.

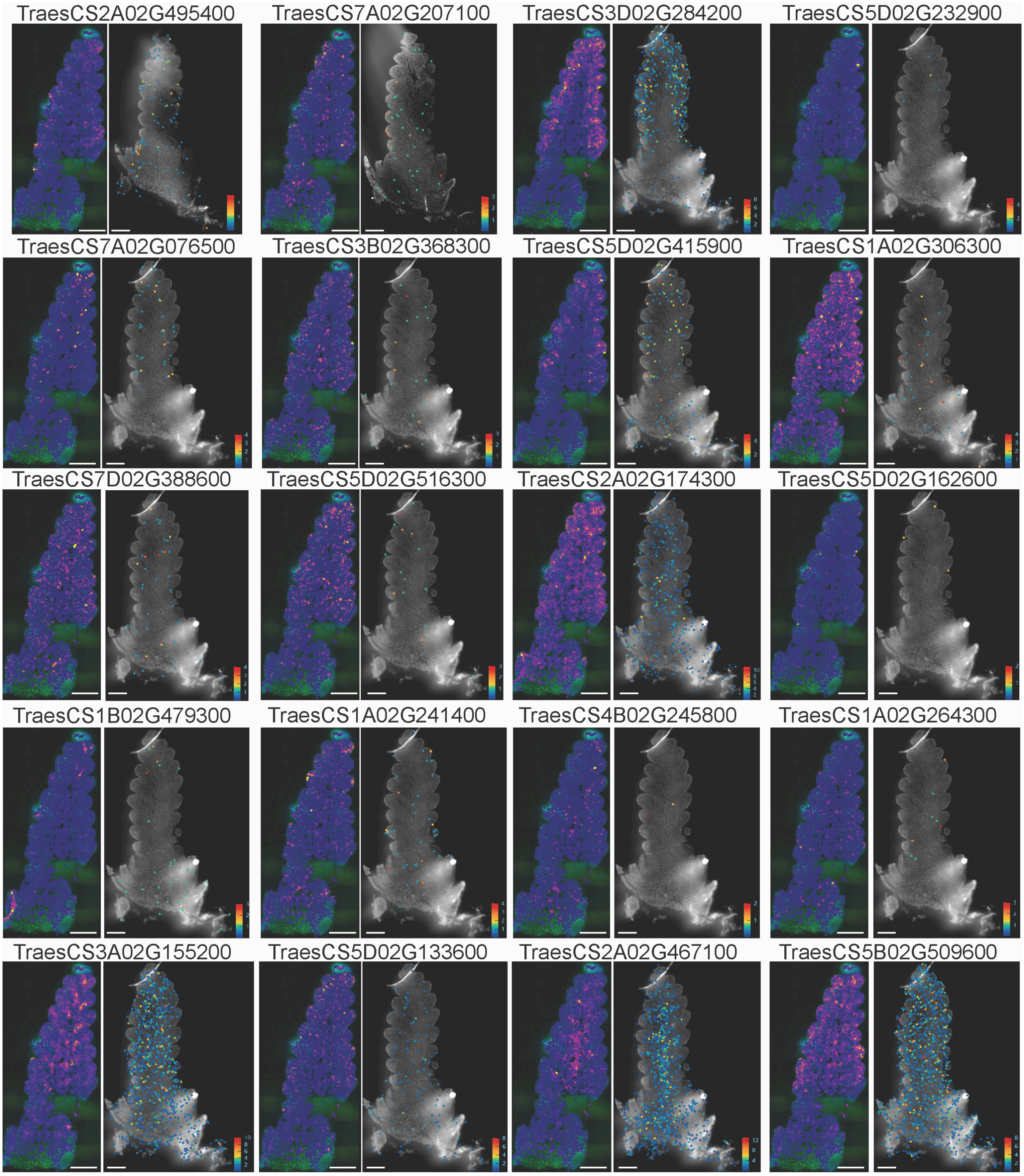

Supplementary Figure 13. Spatial expression comparison of 20 genes randomly selected from the MERFISH panel at the LP stage. Random selection was performed using Excel's RAND() function with fixed values to ensure reproducibility. Stereo-seq maps (right image) show aggregated expression of homeologs to match the non–homeolog-specific methodology of MERFISH (*21*) (left image).

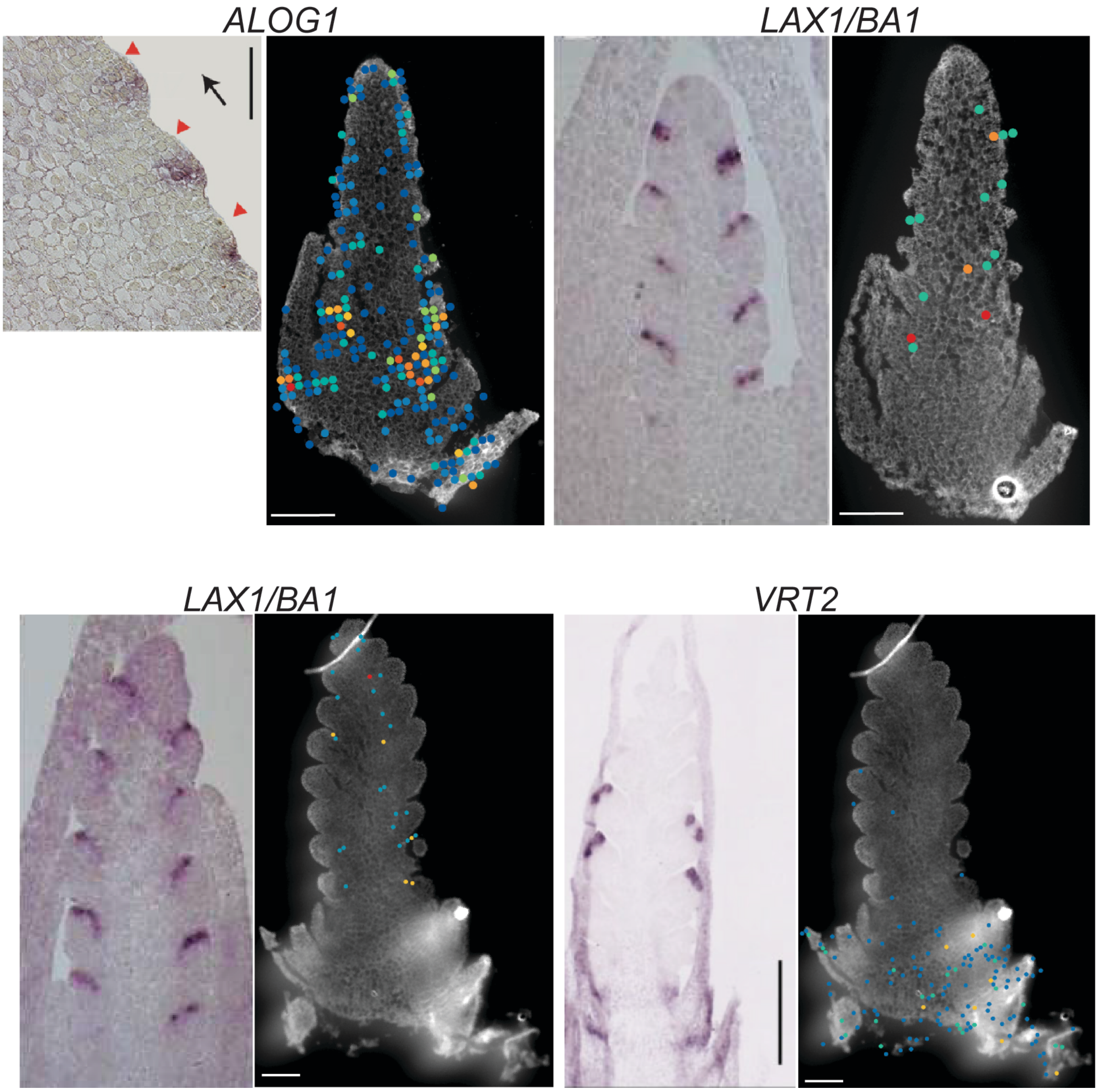

Supplementary Figure 14. Validation of genes with previously published *in-situ* hybridization results. Comparison of gene expression profiles for DR and LP between our spatial transcriptomics dataset (right) and previously published *in-situ* hybridization data (left), showing consistent spatial localization. Genes including *ALOG1* (*3*), *LAX1/BA1*(*77*), and *VRT2* (*18*). Scale bars, 200 μm.

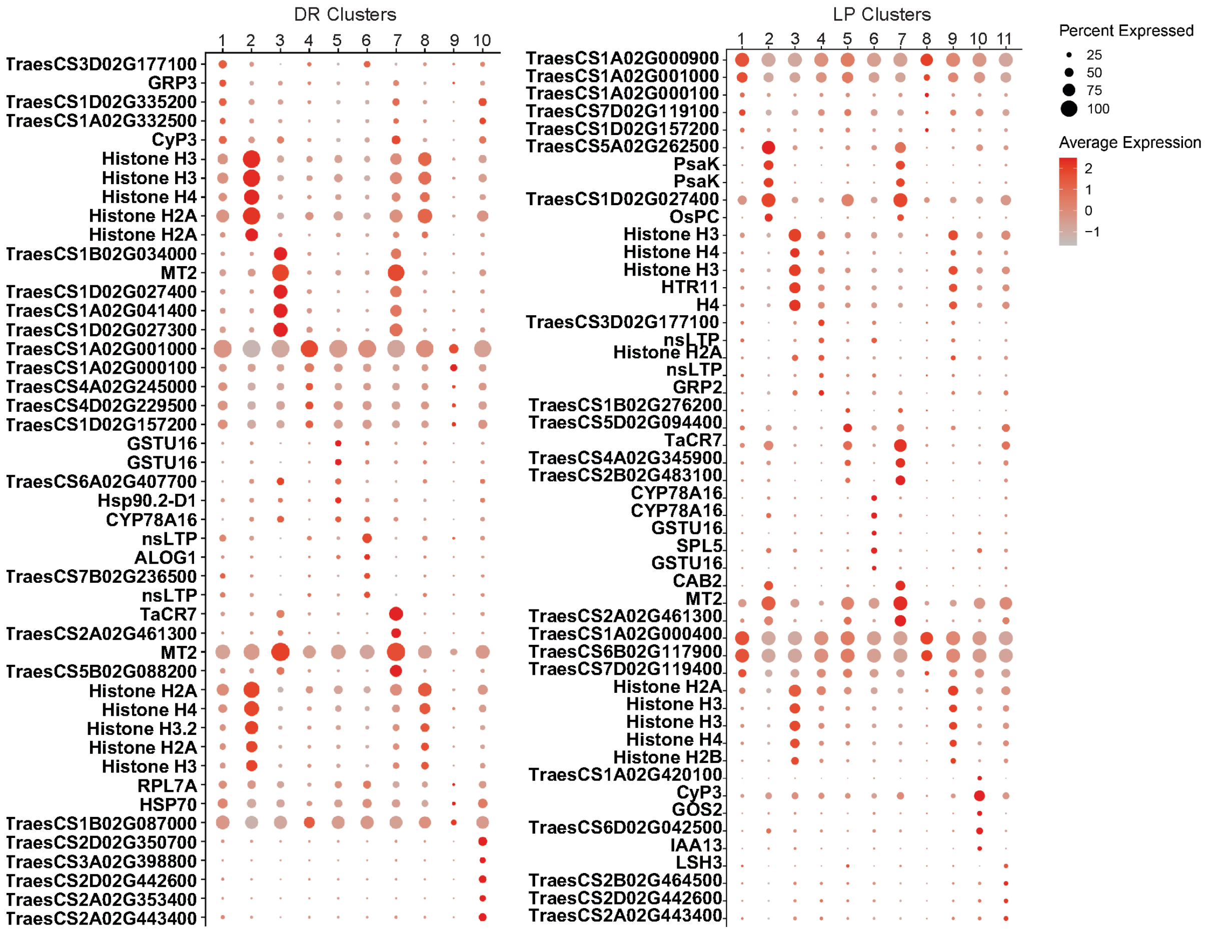

Supplementary Figure 15. Top marker genes. Dot plots displaying the top five marker genes per cluster for both DR and LP. Dot size represents the proportion of bins expressing each gene; color indicates average expression level.

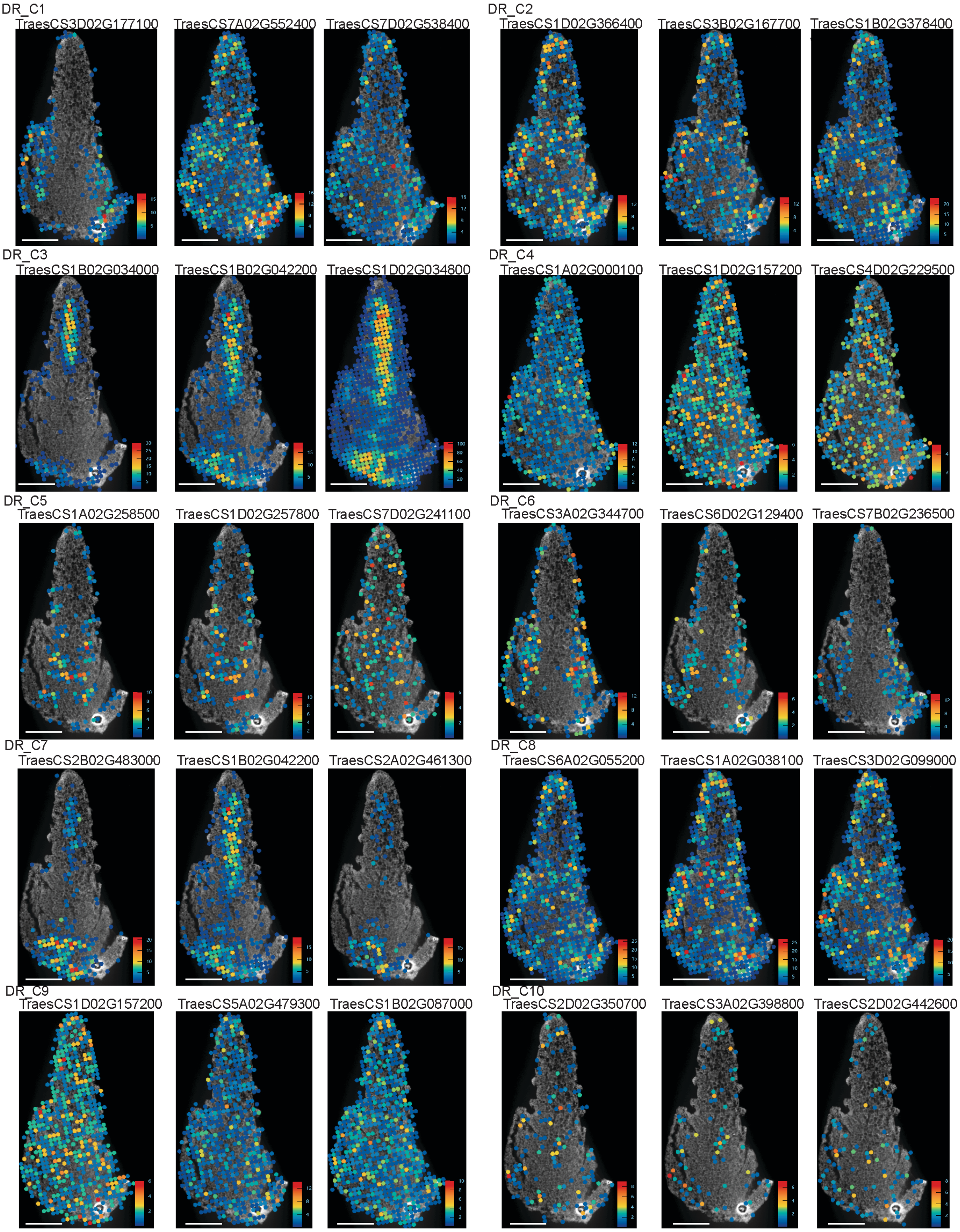

Supplementary Figure 16. Marker genes' spatial expression profiles at DR stage. Spatial gene expression profiles showing three representative marker genes from each cluster at DR stage. Scale bars, 200 μm.

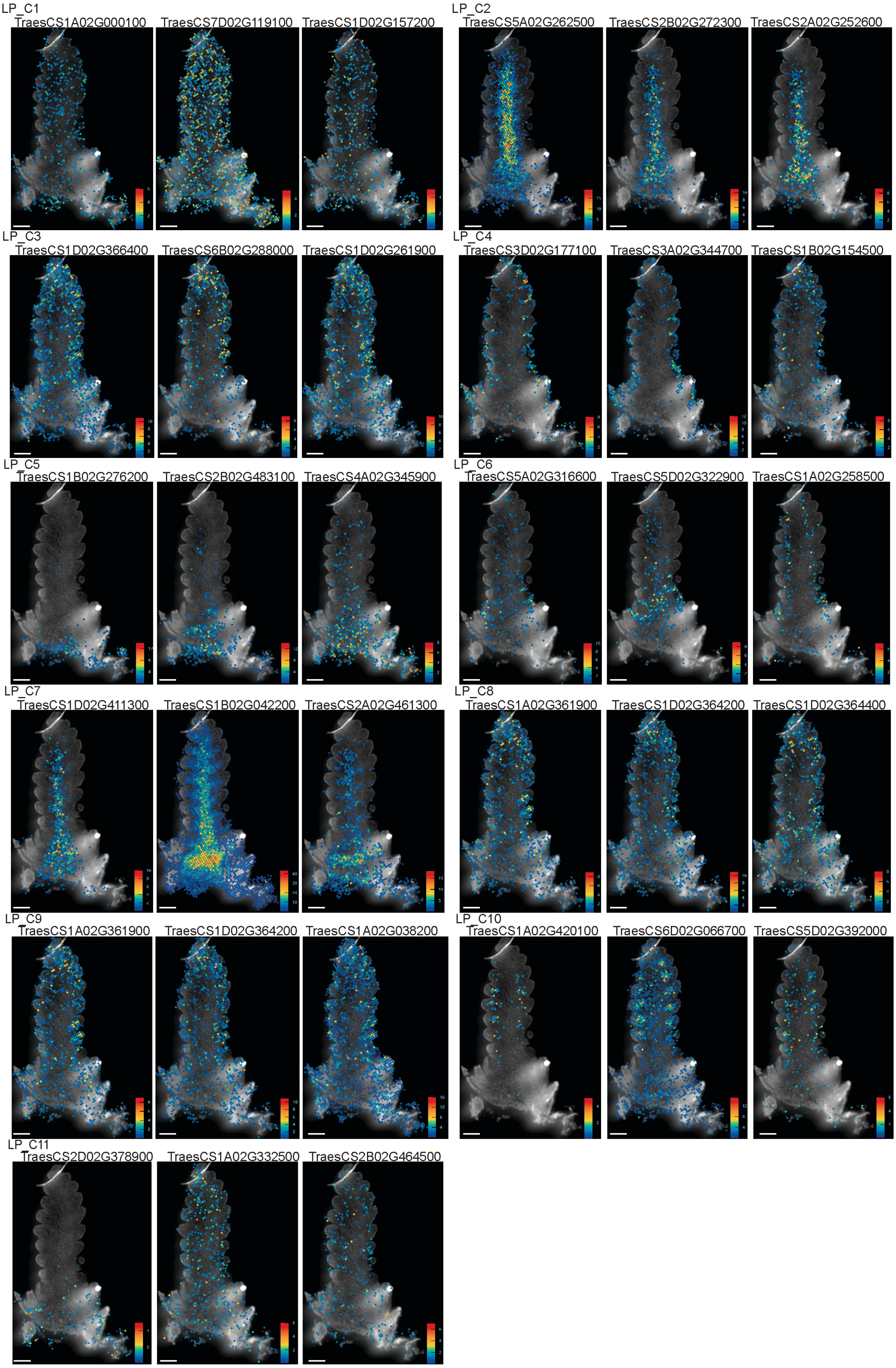

Supplementary Figure 17. Marker genes' spatial expression profiles at LP stage. Spatial gene expression profiles showing three representative marker genes from each cluster at LP stage. Scale bars, 200 μm.

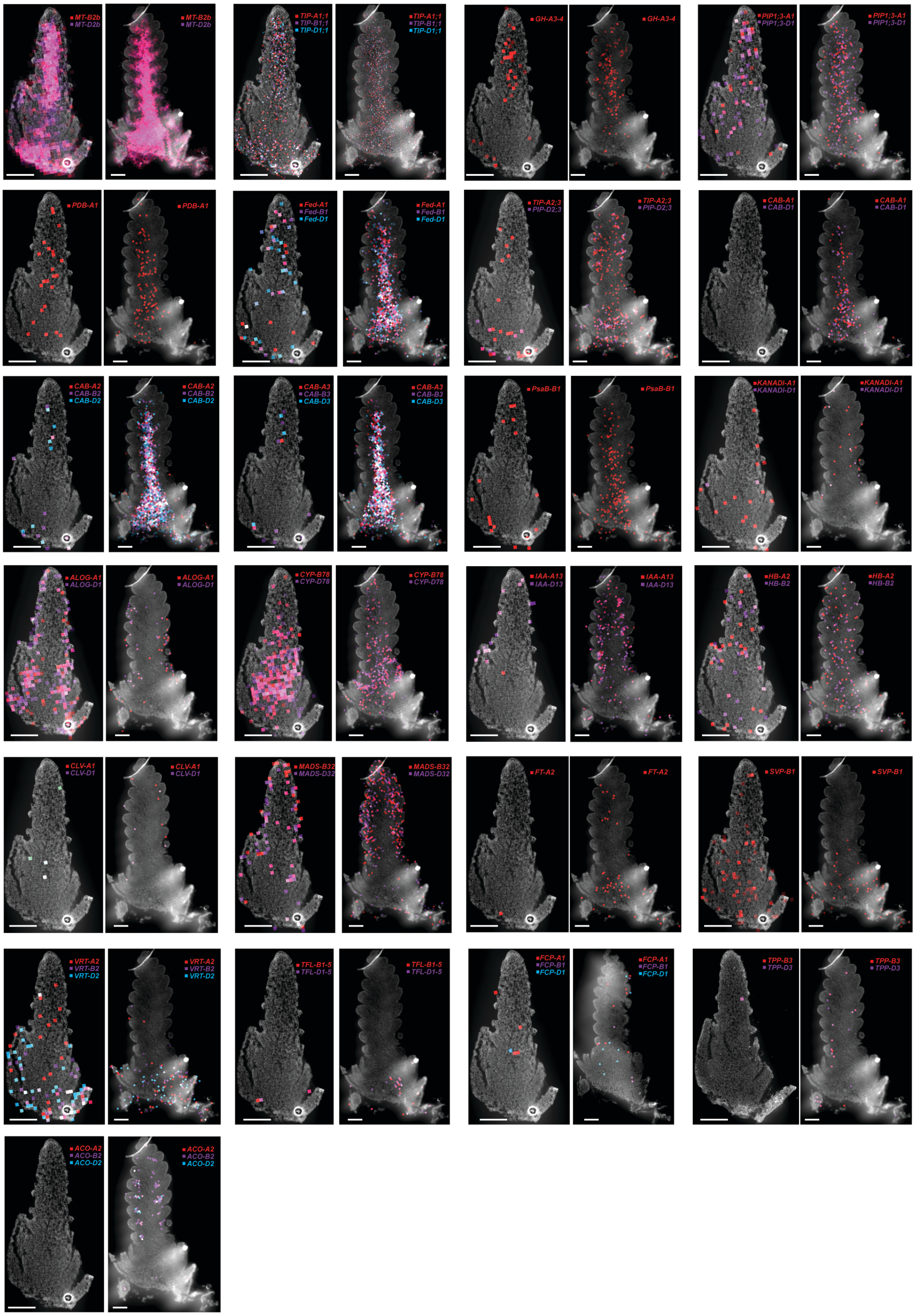

Supplementary Figure 18. Additional gene expression profiles. Expression patterns of additional genes of interest not shown in main figures. Representative images of *IAA13* (Fig. 3), *CAB-D2* (Fig. 5) and *GH3-4* (Fig. 6) are also shown in main figures in comparison to other genes/homeologues. Scale bars, 200 µm.

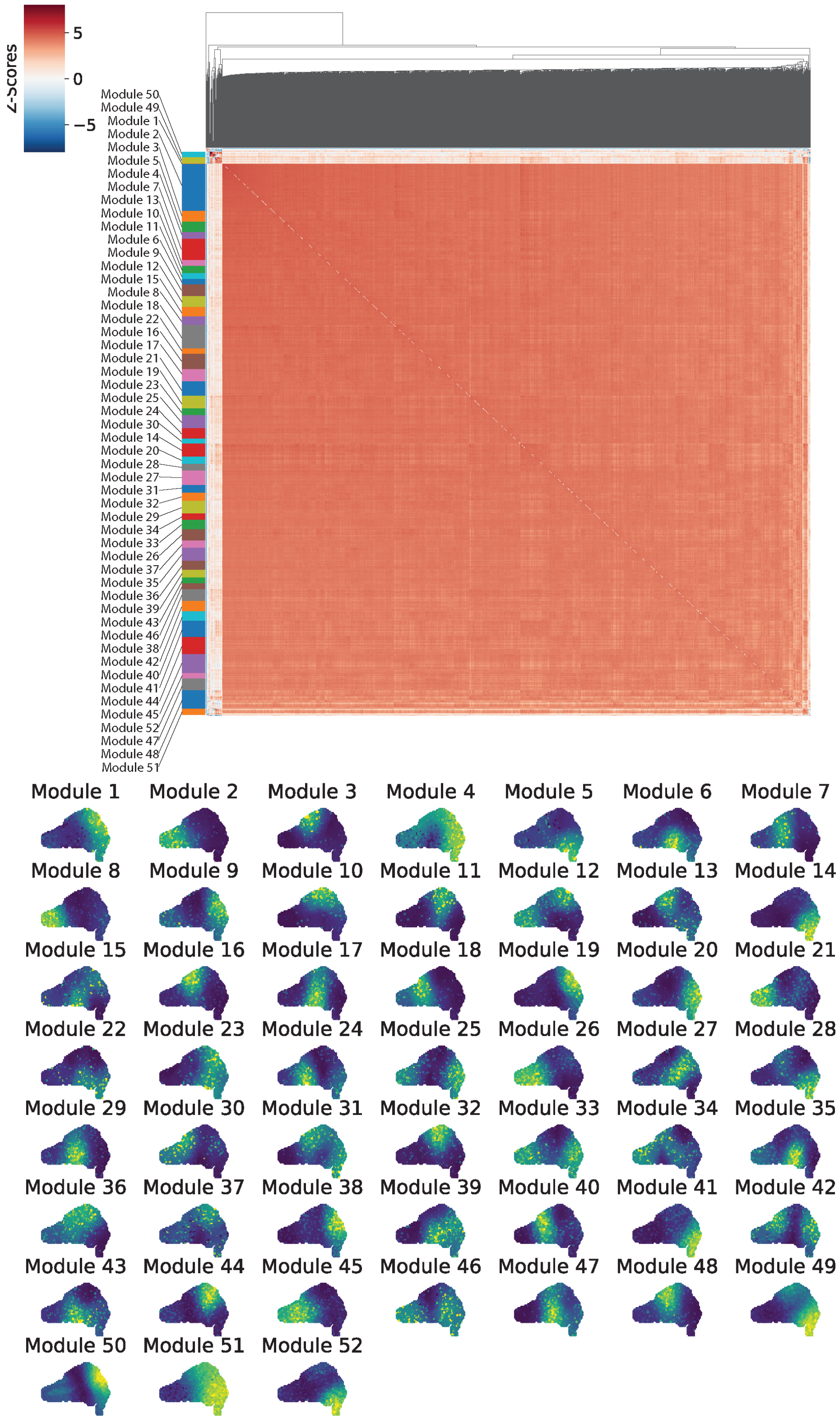

Supplementary Figure 19. Co-expression network in DR stage sections. Visualization of co-expression modules derived from genes expressed in the DR stage. Modules showed limited separation, likely due to the relatively homogenous gene expression across the tissue. This is consistent with the developmental context, as the DR stage represents an earlier, less differentiated phase of inflorescence development.

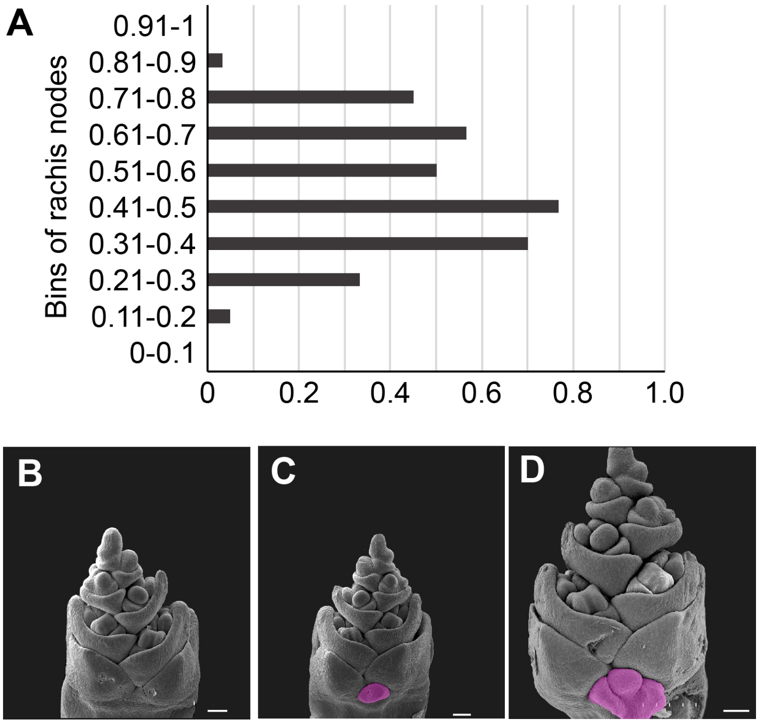

Supplementary Figure 20. SEM and paired spikelet distribution in *ps3* mutant compared to WT. (**A**) Histogram showing the relative frequency of rachis node bins producing paired spikelets in *ps3* spikes (*n* = 10). Y-axis indicates relative vertical position along the spike (normalized from base [0] to apex [1]). (**B**) Scanning electron microscopy (SEM) images of WT inflorescence. (**C-D**), SEM images of *ps3* inflorescences with paired spikelet positions highlighted in magenta. Scale bars, 100 µm.

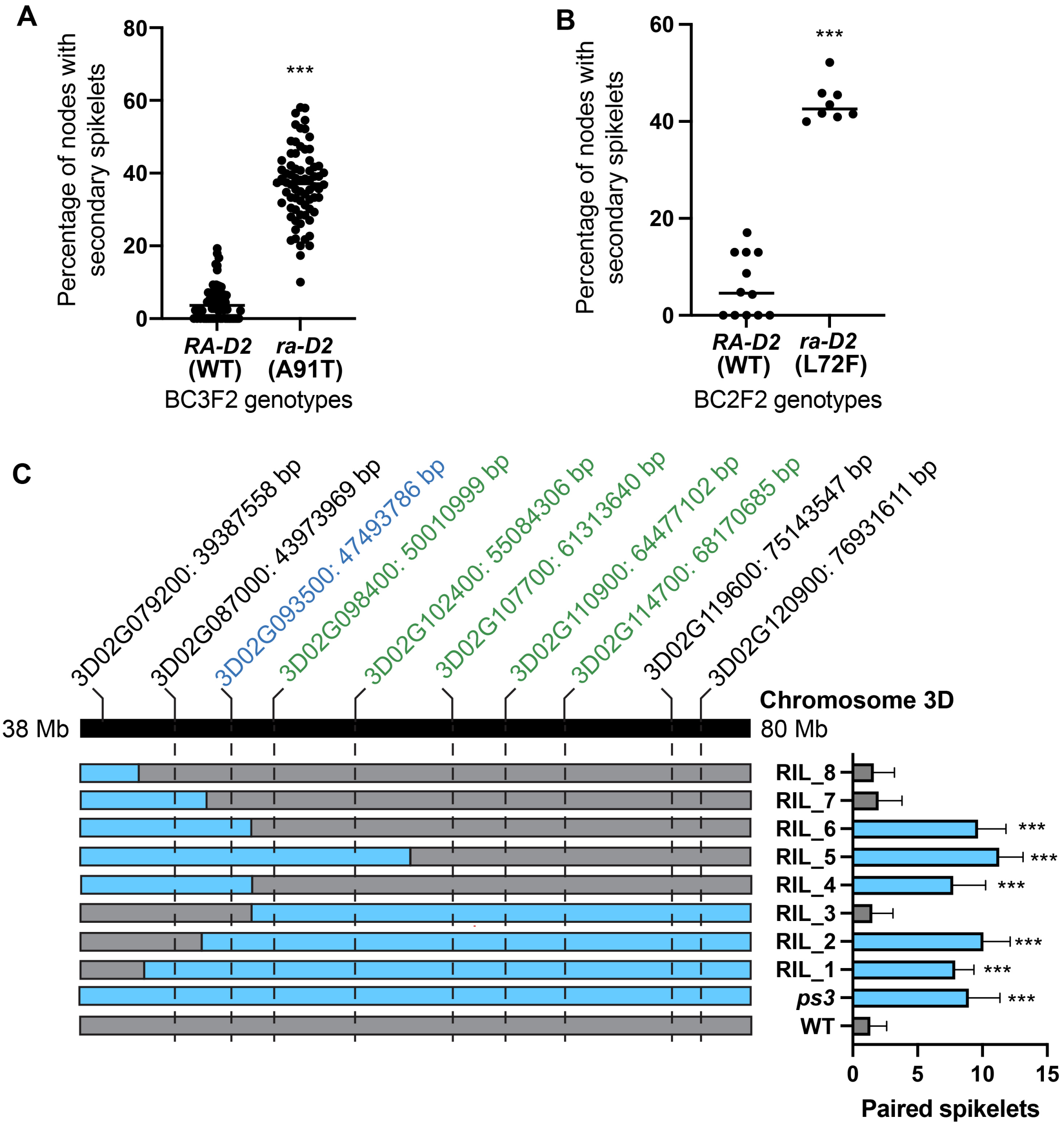

Supplementary Figure 21. Mapping and validation of the *ps3* locus associated with paired spikelets. (**A-B**) Quantification of paired spikelet formation in segregating BC3F2 and BC2F2 populations carrying either WT or mutant alleles of *RA-D2* (A91T and L72F, respectively). Each dot represents a biological replicate. For **A**, n = 66-72 replicate plants, for **B**, n = 8-12 replicate plants ***P< 0.001 (Student's t-test). (**C**) Fine-mapping of *ps3* using recombinant inbred lines (RILs). Genotypes are shown across Chromosome 3D from 38 to 80 Mb using KASP markers (listed above, with physical positions). Blue bars indicate *ps3* mutant-derived segments. The number of paired spikelets in each line is shown to the right. Lines carrying the mutant allele exhibit significantly increased paired spikelets. 6-12 replicate plants were analysed per line (***P < 0.001, Dunnett's test).

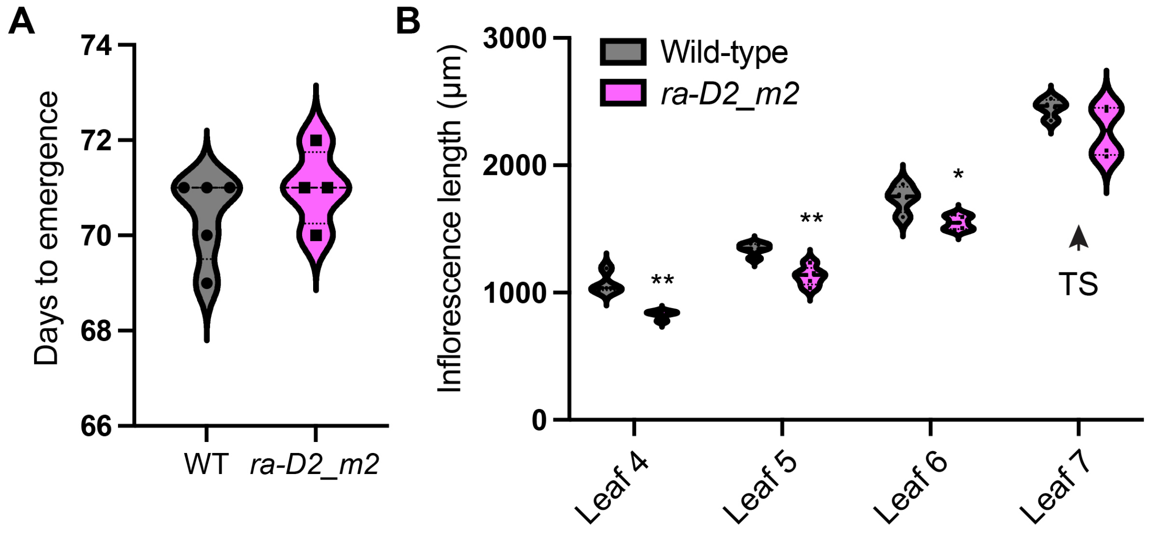

Supplementary Figure 22. Developmental analysis of *CAD0289* (*ra-D2_m2*) mutant. (**A**) Days to emergence are not significantly altered in *ra-D2_m2* (n = 4) compared to WT (n = 5). (**B**) Inflorescence length measured at successive leaf stages (leaf 4 to leaf 7, n = 4-5 replicates per stage) reveals reduced growth in *ra-D2_m2* during early stages, with partial recovery at terminal spikelet (TS) stage. Asterisks indicate statistical significance (*P < 0.05, **P < 0.01; Student's t-test).

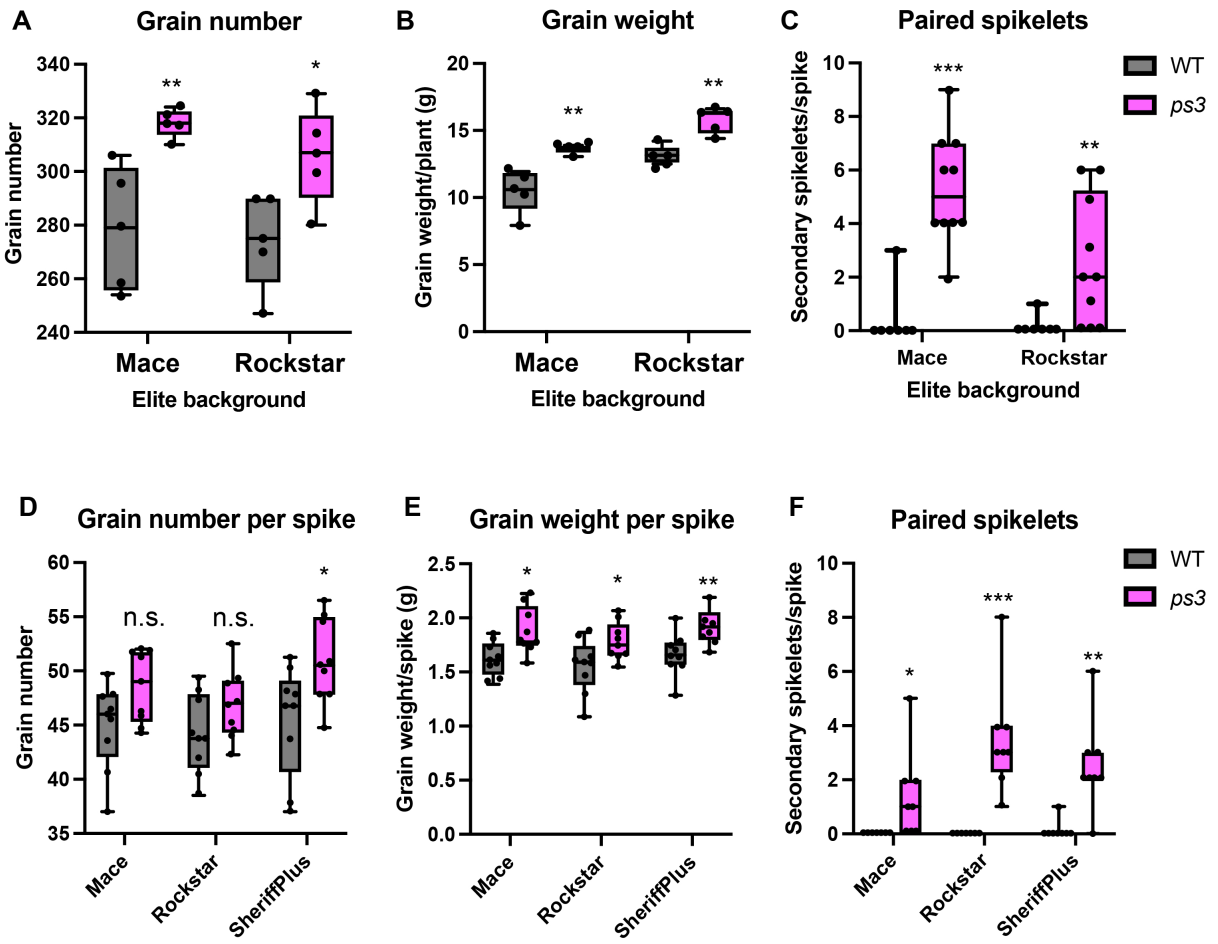

Supplementary Figure 23. Yield-related traits of *ps3* introgression lines in elite backgrounds. (**A-C**) Glasshouse data showing grain number per plant (**A**), grain weight per plant (**B**), and paired spikelets (**C**) in wild type (WT) and *ps3* lines of Mace and Rockstar backgrounds. (**D-F**) Field data showing grain number per spike (**D**), grain weight per spike (**E**), and paired spikelets (**F**) in WT and *ps3* lines of Mace, Rockstar, and Sherriff backgrounds. Box plots show median, interquartile range, and whiskers (min–max), with individual biological replicates plotted as dots. For glasshouse grown plants, n = 5 replicate plants (**A to B**) and 10 spikes (**C**). For field grown plants, data are from 9 replicate spikes harvested from 3 plots of replicate randomised blocks. Significance was determined by Student's t-test (*P < 0.05; **P < 0.01; ***P < 0.001; ****P < 0.0001; ns, not significant).

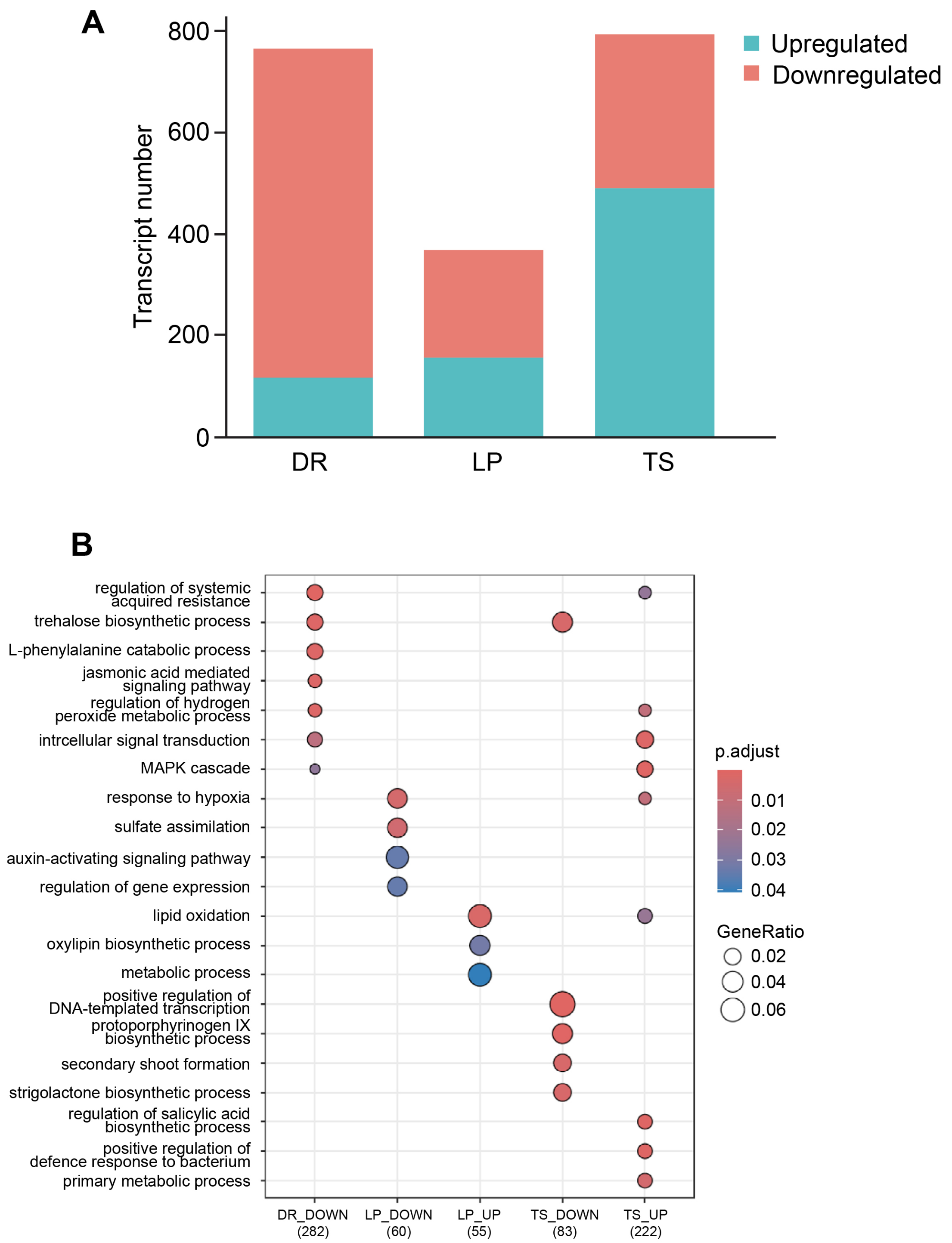

Supplementary Figure 24. Differentially expressed transcripts (DETs) and GO enrichment in *ps3* RNA-seq. (**A**) Bar plot showing the number of upregulated (cyan) and downregulated (salmon) transcripts in *ps3* relative to WT at three developmental stages: DR, LP, and TS. (**B**) Gene Ontology (GO) enrichment analysis of differentially expressed transcripts (DETs). Selected enriched biological processes are shown for each group (DR_DOWN, LP_DOWN, LP_UP, TS_DOWN, TS_UP). Dot size represents gene ratio, and color indicates adjusted P-value.

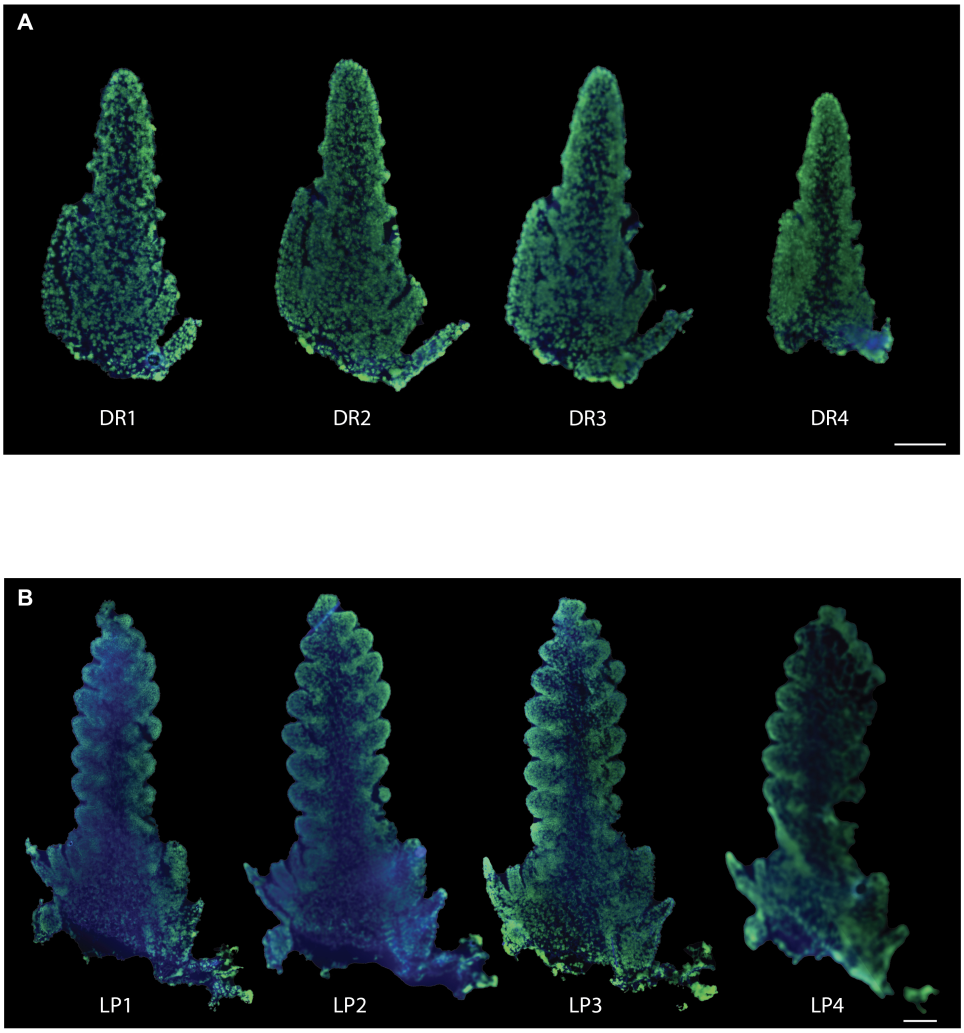

Supplementary Figure 25. Histological staining of wheat inflorescence tissue sections used for Stereo-seq. DR1–DR4 (**A**) and LP1–LP4 (**B**) tissue sections adhered to the surface of the Stereo-seq chip and stained to visualize nuclei and cell walls. Sections were incubated at 37°C for 2 minutes, fixed in methanol at −20°C for 30 minutes, and stained with Qubit ssDNA stain (for nuclei; green) and Fluorescent Brightener 28 (for cell walls; blue). Scale bar = 200 µm.

Table S10: Oligonucleotide sequences for KASP assays of *RA-D2* mutations

Gene ID	Allele	Allele-specific sequence [RO/AO]	Common sequence
3D02G093500	A91T; *ps3*	AGCTCGCTGGCCTACGAG[G/A]	TGGCGCCGACGCAGCCGTA
3D02G093500	L72F; *CAD289*	CCAGCAACGTCACCAAGCTGC[C/T]	GGTGCGGCGGCAGCTCGTT

NB: The prefix for the gene ID is *TraesCS.*

List of Supplementary Tables provided in excel files

Supplementary Table 1: Summary of spatial transcriptomic bin statistics for DR and LP samples.
Supplementary Table 2a: Marker genes for DR spatial clusters.
Supplementary Table 2b: Marker genes for LP spatial clusters.
Supplementary Table 3a: Genes significantly enriched in DR_C1 based on expression patterns.
Supplementary Table 3b: Genes significantly enriched in DR_C2 based on expression patterns.
Supplementary Table 3c: Genes significantly enriched in DR_C3 based on expression patterns.
Supplementary Table 3d: Genes significantly enriched in DR_C4 based on expression patterns.
Supplementary Table 3e: Genes significantly enriched in DR_C5 based on expression patterns.
Supplementary Table 3f: Genes significantly enriched in DR_C6 based on expression patterns.
Supplementary Table 3g: Genes significantly enriched in DR_C7 based on expression patterns.
Supplementary Table 3h: Genes significantly enriched in DR_C8 based on expression patterns.
Supplementary Table 3i: Genes significantly enriched in DR_C9 based on expression patterns.
Supplementary Table 3j: Genes significantly enriched in DR_C10 based on expression patterns.
Supplementary Table 3k: Genes significantly enriched in LP_C1 based on expression patterns.
Supplementary Table 3l: Genes significantly enriched in LP_C2 based on expression patterns.
Supplementary Table 3m: Genes significantly enriched in LP_C3 based on expression patterns.
Supplementary Table 3n: Genes significantly enriched in LP_C4 based on expression patterns.
Supplementary Table 3o: Genes significantly enriched in LP_C5 based on expression patterns.
Supplementary Table 3p: Genes significantly enriched in LP_C6 based on expression patterns.
Supplementary Table 3r: Genes significantly enriched in LP_C8 based on expression patterns.
Supplementary Table 3s: Genes significantly enriched in LP_C9 based on expression patterns.

Supplementary Table 3t: Genes significantly enriched in LP_C10 based on expression patterns.
Supplementary Table 3u: Genes significantly enriched in LP_C11 based on expression patterns.
Supplementary Table 4: Genes expression values for all genes across 10 clusters at DR stage.
Supplementary Table 5: Gene expression values for each gene across 11 clusters at LP stage.
Supplementary Table 6a: Full list of genes assigned to pseudotime expression profiles in DR trajectories.
Supplementary Table 6b: Full list of genes assigned to pseudotime expression profiles in LP trajectories.
Supplementary Table 7: Gene list for modules identified through co-expression network analysis in LP stage.
Supplementary Table 8a: Transcript quantities for differentially expressed transcripts at DR in *ps3*, relative to WT.
Supplementary Table 8b: Transcript quantities for differentially expressed transcripts at DR in *ps3*, relative to WT.
Supplementary Table 8c: Transcript quantities for differentially expressed transcripts at LP in *ps3*, relative to WT.
Supplementary Table 8d: Transcript quantities for differentially expressed transcripts at LP in *ps3*, relative to WT.
Supplementary Table 8e: Transcript quantities for differentially expressed transcripts at TS in *ps3*, relative to WT.
Supplementary Table 8f: Transcript quantities for differentially expressed transcripts at TS in *ps3*, relative to WT.
Supplementary Table 8g: Spatial transcript information for genes differentially expressed in ps3, relative to wild type (Fig. 5I).
Supplementary Table 9: Transcript quantities for leaf-ridge/spikelet boundary genes in WT and *ps3* at double ridge (DR), lemma primordium (LP) and terminal spikelet (TS).
